# Oncogenic *ALK*^*F1174L*^ drives tumorigenesis in cutaneous squamous cell carcinoma

**DOI:** 10.26508/lsa.201900601

**Published:** 2020-04-20

**Authors:** Marco Gualandi, Maria Iorio, Olivia Engeler, André Serra-Roma, Giuseppe Gasparre, Johannes H Schulte, Daniel Hohl, Olga Shakhova

**Affiliations:** 1 Department of Medical Oncology and Hematology, University Hospital Zürich, Zürich, Switzerland; 2 Department of Medical and Surgical Sciences (DIMEC), Medical Genetics Unit, University of Bologna, Bologna, Italy; 3 Department of Pediatric Hematology, Oncology, and Stem Cell Transplantation, Charité—Universitätsmedizin Berlin, Berlin, Germany; 4 German Cancer Consortium (DKTK), Partner Site Berlin and German Cancer Research Center (DKFZ), Heidelberg, Germany; 5 Berlin Institute of Health, Berlin, Germany; 6 Department of Dermatology and Venereology, Hôpital de Beaumont, Lausanne University Hospital Centre, Lausanne, Switzerland

## Abstract

Cutaneous squamous cell carcinoma (cSCC) is the second most common skin cancer characterized by increased mortality. Here, we show for the first time that anaplastic lymphoma kinase (ALK), a receptor tyrosine kinase of the insulin receptor superfamily, plays a pivotal role in the pathogenesis of cSCC. Our data demonstrate that the overexpression of the constitutively active, mutated ALK, *ALK*^*F1174L*^, is sufficient to initiate the development of cSCC and is 100% penetrant. Moreover, we show that cSCC development upon *ALK*^*F1174L*^ overexpression is independent of the cell-of-origin. Molecularly, our data demonstrate that *ALK*^*F1174L*^ cooperates with oncogenic *Kras*^*G12D*^ and loss of *p53*, well-established events in the biology of cSCC. This cooperation results in a more aggressive cSCC type associated with a higher grade histological morphology. Finally, we demonstrate that Stat3 is a key downstream effector of *ALK*^*F1174L*^ and likely plays a role in *ALK*^*F1174L*^-driven cSCC tumorigenesis. In sum, these findings reveal that ALK can exert its tumorigenic potential via cooperation with multiple pathways crucial in the pathogenesis of cSCC. Finally, we show that human cSCCs contain mutations in the *ALK* gene. Taken together, our data identify ALK as a new key player in the pathogenesis of cSCC, and this knowledge suggests that oncogenic ALK signaling can be a target for future clinical trials.

## Abstract (precis)

Here, we show for the first time that anaplastic lymphoma kinase (ALK), a receptor tyrosine kinase of the insulin receptor superfamily, plays a pivotal role in the pathogenesis of cSCC.

## Introduction

Anaplastic lymphoma receptor kinase (ALK) alterations have been identified in several human cancers, including neuroblastoma, glioblastoma, lung cancer, anaplastic large cell lymphoma, and renal cell carcinoma (Hallberg & Palmer, 2013). Most cancer-associated rearrangements in the *ALK* gene are associated with fusions, copy-gain number, or activating *ALK* mutations (Hallberg & Palmer, 2013). In mice, overexpression of the mutated *ALK*^*F1174L*^ gene results in neuroblastoma development (Heukamp et al, 2012). An elevated expression of phosphorylated ALK as well as its ligands, midkine, and pleiotrophin has been found in patients with basal cell carcinoma (BCC) and cSCC (Ning et al, 2013). To investigate the possible role of ALK in the pathogenesis of skin tumors, we overexpressed *ALK*^*F1174L*^ in the epithelial cells in the skin. A number of studies has addressed the cell-of-origin of BCC and cSCC. BCC can arise from the progenitor cells of the interfollicular epidermis, cells in the infundibulum of the hair follicle (HF) (Youssef et al, 2010), and HF stem cells (Grachtchouk et al, 2011). Similarly, compelling evidence suggests that cSCC can also arise not only from interfollicular epidermis but also from the HF stem cells (Lapouge et al, 2011; White et al, 2011; Sanchez-Danes & Blanpain, 2018). Based on these studies, we have decided to overexpress *ALK*^*F1174L*^ in HF stem cells using *Lgr5-CreERT2* (Barker et al, 2007) and *K15-CreERT2* (Morris et al, 2004) mouse lines, and in all basal cells taking advantage of *K5-CrePR1* (Zhou et al, 2002) and *K14-CreERT2* (Vasioukhin et al, 1999) transgenic strains.

## Results and Discussion

**Figure 1.**
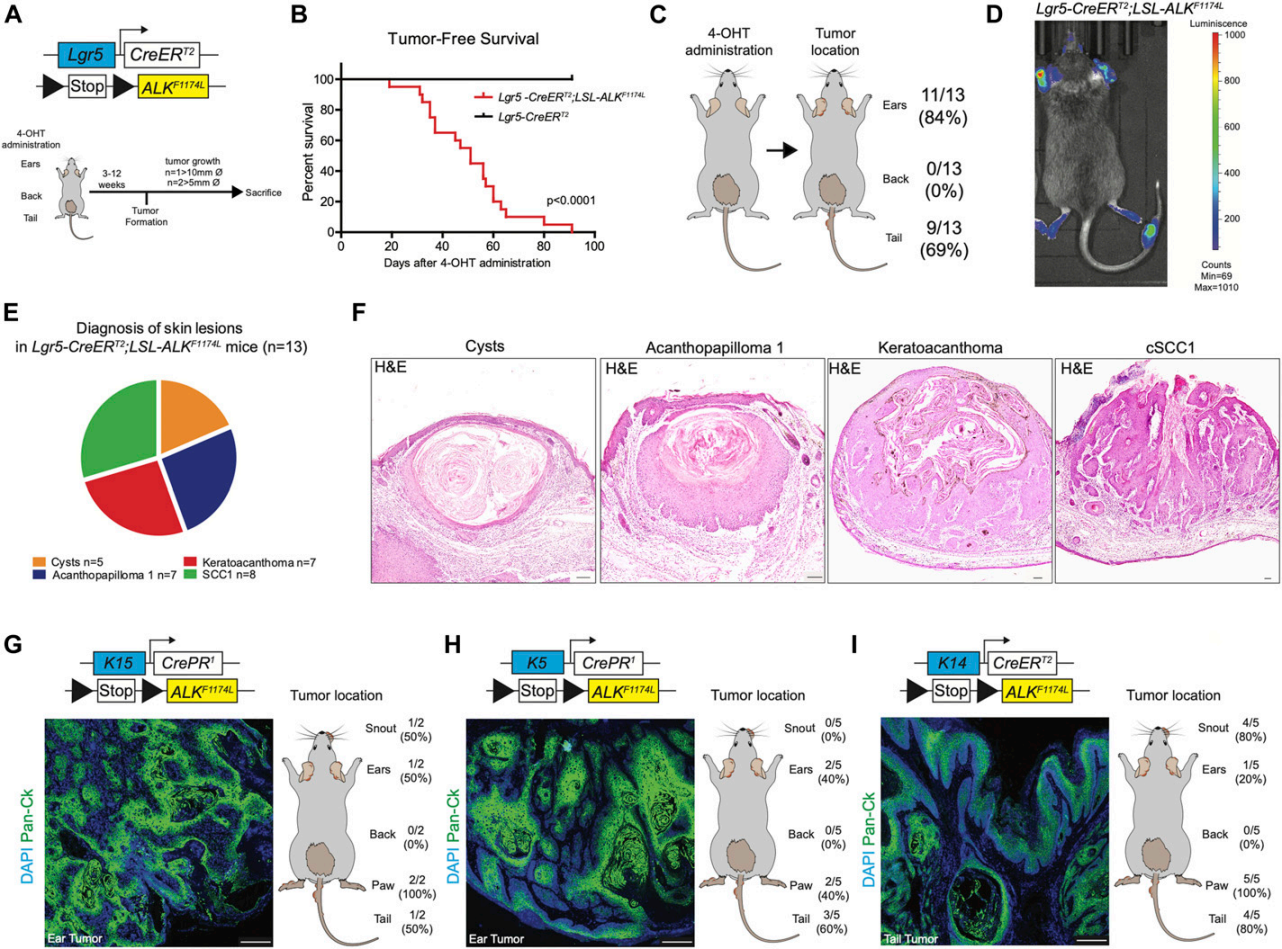
Expression of *ALK*^*F1174L*^ in different skin compartments induces skin lesions and cSCC. **(A)** Graphical representation of *Lgr5-CreER*^*T2*^*;LSL-ALK*^*F1174L*^ genotype and experimental design. Topical application of 4OH-tamoxifen (4-OHT) in ears, back, and tail skin of mice resulted in skin lesion development. Then, mice were euthanized when termination criteria were observed (tumor size and ulceration). **(B)** Tumor-free survival of *Lgr5-CreER*^*T2*^*;LSL-ALK*^*F1174L*^ mice (n = 15, median 47 d) and controls (n = 12). Log-rank (Mantel–Cox) Test *P* < 0.0001, HR 28.12. All mice developed tumors. **(C)** From left to right. Topical administration of 4-OHT and after tumor formation per location. **(D)** Representative picture of in vivo imaging system (IVIS). Analysis of the *LSL-ALK*^*F1174L*^ transgene expression shows strong luminescent signal from the tumors on ears and tail. **(E)** Pie chart representing the number of mice that developed the listed skin lesions out of total skin lesions diagnosed. **(F)** Representative hematoxylin and eosin (H&E) staining of such lesions from the ears. Scale bars = 100 µm. To note, each mouse developed several tumors. **(G, H, I)** Top to bottom. Genotypes, representative pictures of the tumors marked by pan-cytokeratin immune-labeling and tumor distribution per location. To note, because of leakage of the Cre expression, tumor formation was observed when no specific topical administration was performed. Scale bars = 250 µm.

**Figure S1.**
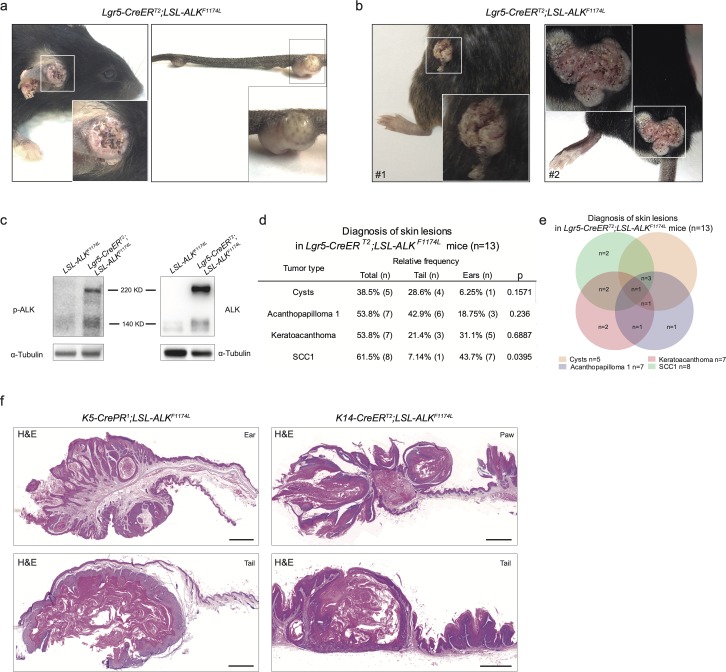
**Overepxression of *ALK^F1174L^* induces skin tumorigenesis independently of the cell of origin. (A)** Representative pictures of ear and tail tumors of *Lgr5-CreER*^*T2*^*;LSL-ALK*^*F1174L*^ mice. **(B)** Representative pictures of mice that developed tumors on the back skin after wounds (due to fighting) were observed. Because of the interference of wounding on normal skin homeostasis, these mice have been withdrawn from experiments. **(C)** Western blot for p-ALK and α-tubulin as loading control, in *Lgr5-CreER*^*T2*^*;LSL-ALK*^*F1174L*^ ear tumor protein lysate and control ear from sibling. **(D, E)** Evaluation of the skin lesions of *Lgr5-CreER*^*T2*^*;LSL-ALK*^*F1174L*^ mice. **(D)** Column “Total” represents the percentage of mice that developed the corresponding diagnosed lesion. The *P*-value has been calculated by Fisher’s exact test in contingency table comparing the proportion of the corresponding skin lesion out of the total lesions per location (tail or ear). **(E)** Venn diagram representing the distribution of the different skin lesions in the mice. **(F)** Representative hematoxylin and eosin (H&E) staining of *K5-CrePR*^*1*^*;LSL-ALK*^*F1174L*^ and *K14-CreER*^*T2*^*;LSL-ALK*^*F1174L*^ skin lesions. Scale bar = 1 mm.

We induced the expression of *ALK*^*F1174L*^ via topical administration of 4-hydroxytamoxifen (4-OHT) on the shaved back skin as well as on the ears and tails (Fig 1A). 100% of *ALK*^*F1174L*^ *Lgr5-CreERT2* mice developed skin lesions and had to be euthanized at the latest 4 mo after 4-OHT induction (Figs 1B and S1A). Skin lesions became apparent after 3 wk after transgene activation. Whereas 83% (11/13 mice) of *ALK*^*F1174L*^ *Lgr5-CreERT2* mice developed lesions in the ears and 69% (9/13 mice) in the tail, no abnormalities were seen in the back skin (Fig 1C). However, skin lesions on the back skin were occasionally observed in several *ALK*^*F1174L*^ overexpressing mice carrying fight wounds (Fig S1B). It is widely accepted that epithelial cancers arise as a result of a multistep process involving tumor initiation, promotion, and progression (Hennings & Boutwell, 1970; Abel et al, 2009). The fact that skin wounding in combination with other inducing agents has been previously demonstrated to promote skin carcinogenesis (Hoste et al, 2015) suggests that whereas *ALK*^*F1174L*^ overexpression alone is sufficient to drive tumor formation in ear and tail skin, it might require an additional promoting treatment in the back skin. We nevertheless excluded those mice from further analysis because our study focused on the dissection of the role of *ALK*^*F1174L*^ overexpression in the context of skin homeostasis. ALK overexpression was confirmed using Western blot with anti-pALK antibodies (Fig S1C). The presence of the *luciferase* (*luc*) reporter gene in the *ALK* transgene allowed us to monitor tumor development using IVIS imaging system (Heukamp et al, 2012) (Fig 1D). Based on the histological examination (Gleich et al, 2016), we distinguished four types of skin lesions, including cysts (n = 5 mice), acanthopapilloma (AP) (n = 7 mice), keratoacanthoma (KA) (n = 7 mice), and cSCC type 1 (n = 8 mice) (Figs 1E and F and S1D and E). Similarly, the targeted expression of *ALK*^*F1174L*^ using another HF stem cell–specific *Cre* line, *K15-CrePR1* (Morris et al, 2004), resulted in cSCC development (Fig 1G). Moreover, the crossings of *ALK*^*F1174L*^ mice with *K5-CrePR1* (Zhou et al, 2002) and *K14-CreERT2* (Vasioukhin et al, 1999) lines gave rise to skin lesions strikingly resembling those present in *Lgr5-CreERT2* and *K15-CrePR1* lines as assessed with hematoxylin/eosin staining as well as with an immunostaining for pan-cytokeratin (pan-Ck) (Figs 1H and I and S1F). We conclude that *ALK*^*F1174L*^ expression alone is sufficient to drive tumor initiation, and it induces cSCC independently of the cell-of-origin.

**Figure S2.**
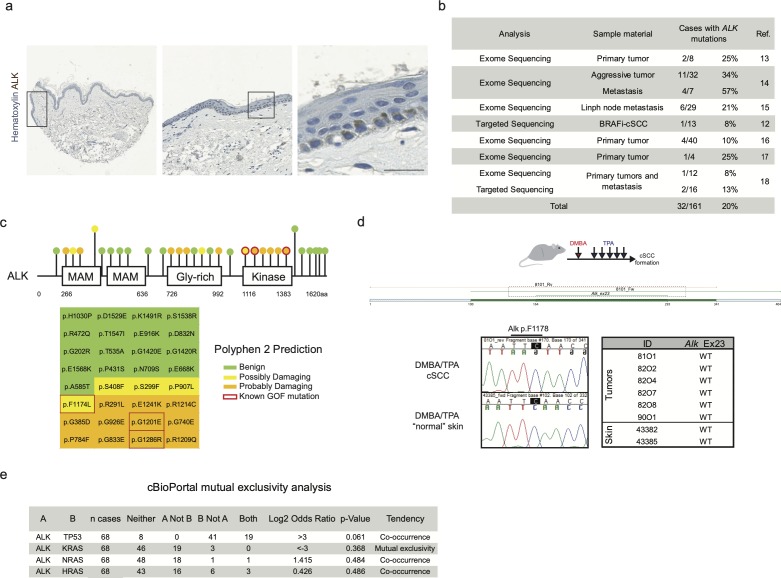
**Characterization of the expression and mutations in human and mouse *ALK* genes. (A)** Immunohistochemistry of a human skin sample immune-labeled with anti-ALK antibody shows positivity among cells of the basal layer of the epidermis. Scale bar = 25 µm. **(B, C)** Metanalysis of *ALK* mutations from different cohorts of cSCC patients. **(B)** number of cases with mutation in the *ALK* gene. **(C)** Graphical representation of the *ALK* mutations and PolyPhen 2 prediction. Red rectangles point out known gain of function (GOF) mutations. **(D)** Graphical representation of DMBA/TPA skin carcinogenesis model. Exome 23 of *Alk* have been sequenced in six tumors and two “normal” skin arising from induced skin areas. No mutations in *Alk* pF1178 have been observed (mouse residue of the human F1174). **(E)** Analysis of frequency of co-occurrence of mutations in *ALK*, *TP53*, and *RAS* genes. Co-occurrence or mutual exclusivity has been assessed thanks to the cBioPortal database. *P*-value is derived from one-sided Fisher’s exact test.

Next, we examined the expression of ALK protein in the normal human skin. ALK expression was readily detected in virtually all basal keratinocytes (Fig S2A). Given the fact that ALK expression in humans has been described in both tumor types, BCC and cSCC, it was surprising that we did not observe any signs of BCC development in our mouse model. To investigate whether mutations in *ALK* gene are present in the human cSCC samples, we performed the analysis of publicly accessible data resources. Cohen et al (2015) described the landscape of mutations detected in cSCC diagnosed in patients undergoing BRAF inhibitor therapy for advanced metastatic melanoma and among other mutations, they have detected the *ALK*^*F1174L*^ mutation (1/29 patients). This prompted us to investigate the frequency of *ALK* mutations in primary and metastatic human cSCC, and we focused on analysis of human cSCC using exome sequencing (Fig S2B) (Durinck et al, 2011; Lee et al, 2014; Pickering et al, 2014; Li et al, 2015; Yilmaz et al, 2017; Inman et al, 2018). Of 161 human cSCC cases analyzed, we have identified 32 cases (20%) carrying mutations in the *ALK* gene. PolyPhen-2 software was used to predict damaging effects of identified *ALK* mutations (Fig S2C) (Adzhubei et al, 2010). Whereas nearly 50% of cases predicted no effect on the *ALK* gene function, others were characterized as potentially harmful mutations. Of note, in addition to the p.F1174L mutation identified in cSCC occurring in the melanoma patient (Cohen et al, 2015), two additional cases harbored the p.G1201E (Murugan & Xing, 2011) and p.G1286R (Bresler et al, 2014) mutations, which are known *ALK* gain-of-function mutations (Fig S2C). We also checked for the presence of mutations in the *Alk* gene in the previously described mouse models of cSCC (Nassar et al, 2015). Two mutations were described, namely, the p.C787S and the p.C1012S, which, however, did not correlate with Alk expression (Nassar et al, 2015).

To directly assess whether the *Alk*^*F1178L*^ (orthologue to *ALK*^*F1174L*^) mutation is present in mouse cSCC, we have sequenced exon 23 of the *Alk* gene from six independent cSCC induced by a combination of DMBA (7,12-dimethylbenzanthracene) and TPA (12-*O*-tetradecanoylphorbol-13-acetate) treatment (Fig S2D). None of the tumors exhibited mutations in the *Alk* gene. This could be due to the fact that all mouse DMBA/TPA tumors are induced on the back skin, a location devoid of skin lesions in *ALK*^*F1174L*^ overexpressing mice. Because DMBA/TPA–induced tumors are predominantly driven by oncogenic mutations in RAS signaling, an alternative explanation may be that ALK and RAS are mutually exclusive drivers of skin tumorigenesis. To test this hypothesis, we have analyzed the co-occurrence and overall frequency of *ALK* and *RAS* mutations in human cSCC using cBio Portal (Fig S2E). Among all *RAS* genes analyzed, we have observed that mutations in *ALK* gene appear to be mutually exclusive with mutations in *KRAS* gene (68 cases analyzed and 0 cases display co-occurrence). Mutations in *HRAS* and *NRAS* genes co-occurred with mutations in *ALK* in some cases (68 cases analyzed, 1 case co-occurrence of *NRAS* and *ALK* in the same patient, and 3 cases show co-occurrence of *ALK* and *HRAS* mutations).

**Figure S3.**
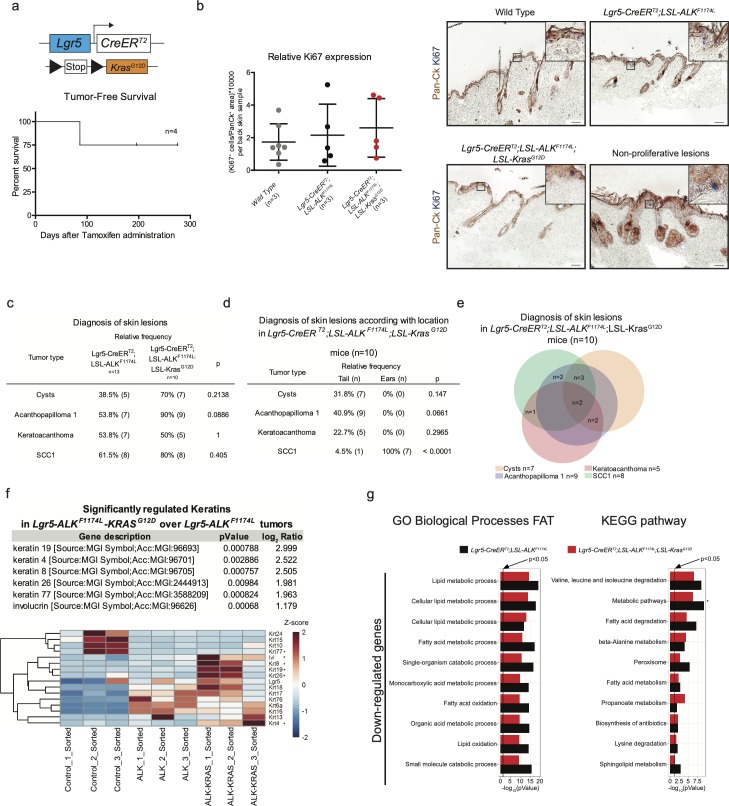
***ALK^F1174L^* cooperates with *Kras^G12D^* to drive cSCC tumorigenesis. (A)** Graphical representation of *Lgr5-CreER*^*T2*^*;LSL-Kras*^*G12D*^ genotype and tumor-free survival (n = 4). **(B)** Quantification of proliferating cells in back skins from *wild-type*, *Lgr5-CreER*^*T2*^*;LSL-ALK*^*F1174L*^ and *Lgr5-CreER*^*T2*^*;LSL-ALK*^*F1174L*^*;LSL-Kras*^*G12D*^ mice and representative immunohistochemistry carried out using pan-cytokeratin (Pan-Ck) an Ki67 antibodies. Anagen hair follicles have been excluded. Data showed as mean ± SD (one-way analysis of variance, *P* = 0.6517). Scale bars = 50 µm. **(C, D, E)** Evaluation of the skin lesions of *Lgr5-CreER*^*T2*^*;LSL-ALK*^*F1174L*^*;LSL-Kras*^*G12D*^ mice. **(C)** The *P*-value has been calculated by Fisher’s exact test in contingency table comparing the proportion of the corresponding skin lesion out of the total lesions per location (tail or ear). **(D)** The *P*-value has been calculated by Fisher’s exact test in contingency table comparing the proportion of *Lgr5-CreER*^*T2*^*;LSL-ALK*^*F1174L*^ and *Lgr5-CreER*^*T2*^*;LSL-ALK*^*F1174L*^*;LSL-Kras*^*G12D*^ mice that developed the corresponding skin lesion out of total mice per group. **(E)** Venn diagram representing the distribution of the different skin lesions in the mice. **(F)** Above: table showing the RNA relative expression of significantly mutated keratins between *Lgr5-CreER*^*T2*^*;LSL-ALK*^*F1174L*^ versus *Lgr5-CreER*^*T2*^*;LSL-ALK*^*F1174L*^*;LSL-Kras*^*G12D*^ sorted tumor cells. Below: heat map showing all keratins mutated between sorted ear keratinocytes versus *Lgr5-CreER*^*T2*^*;LSL-ALK*^*F1174L*^ and *Lgr5-CreER*^*T2*^*;LSL-ALK*^*F1174L*^*;LSL-Kras*^*G12D*^ sorted tumor cells. **(G)** Significantly mutated biological processes and KEGG signaling pathways between *Lgr5-CreER*^*T2*^*;LSL-ALK*^*F1174L*^ and *Lgr5-CreER*^*T2*^*;LSL-ALK*^*F1174L*^*;LSL-Kras*^*G12D*^ tumors. Horizontal columns represent the *P*-value of the analysis of the down-regulated genes from RNA-seq transcriptional profiling of sorted tumor cells versus normal keratinocytes. Metabolic pathways that is marked by * is significantly changed also in the analysis of transcriptomes of *Lgr5-CreER*^*T2*^*;LSL-ALK*^*F1174L*^ versus *Lgr5-CreER*^*T2*^*;LSL-ALK*^*F1174L*^*;LSL-Kras*^*G12D*^ tumor cells.

Furthermore, we ruled out the possibility that the back skin of *ALK*^*F1174L*^ *Lgr5-CreERT2* mice contained early lesions, yet not visible to the naked eye. To this aim, we performed thorough histological and immunohistochemical examination with anti-Ki67 antibodies and quantified the proliferation index of keratinocytes identified by pan-Ck staining (Fig S3B). Even though some HF appeared dysplastic, no increased proliferation was observed.

**Figure 2.**
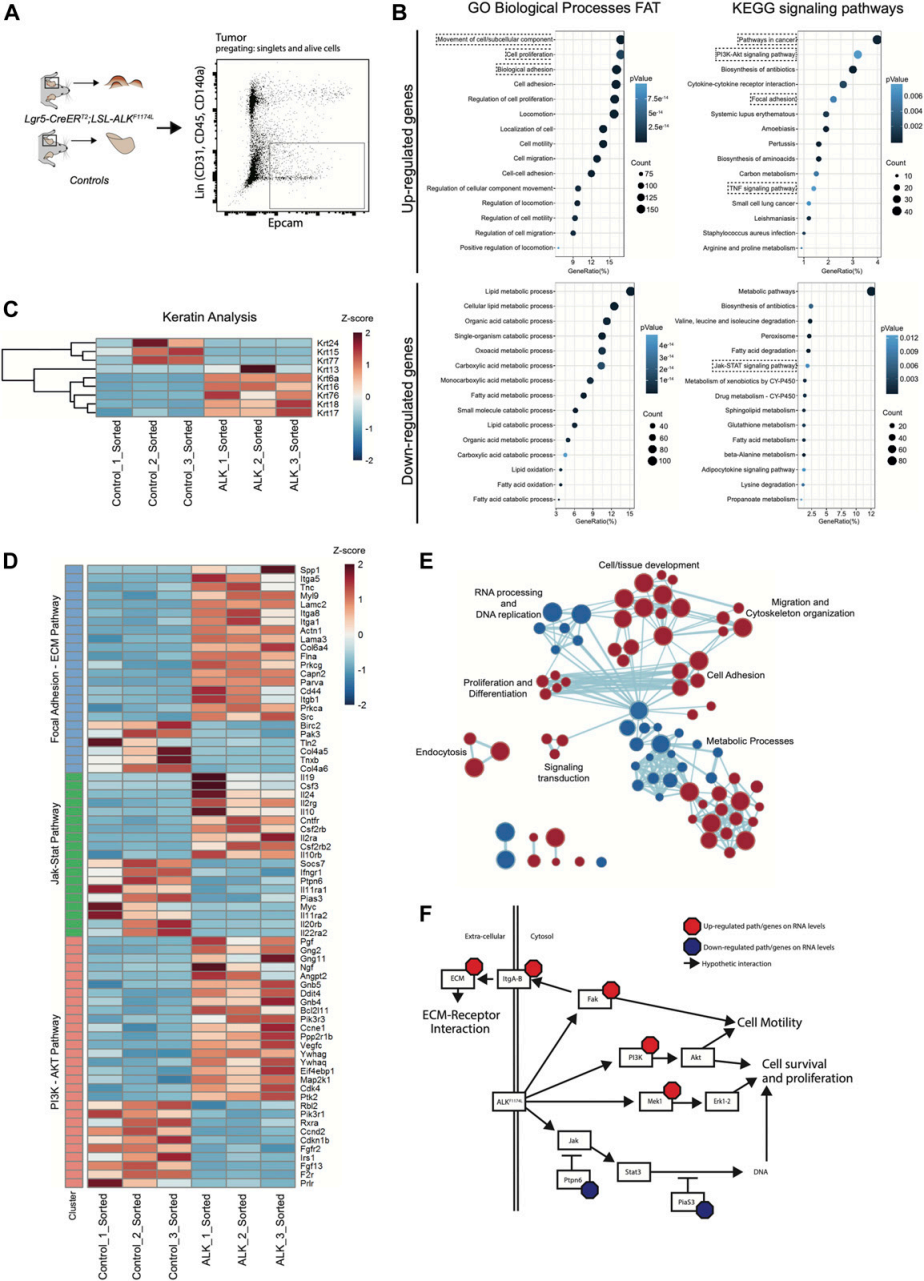
ALK^F1174L^–driven tumorigenesis relied in bona fide, via PI3K-AKT/focal adhesion–ECM receptor interaction pathways. **(A)** Ear tumor from *Lgr5-CreER*^*T2*^*;LSL-ALK*^*F1174L*^ mice and ears from control mice were prepared as a single-cell suspension. Tumor cells and keratinocytes were isolated by FACS for EpCAM expression e-negative selected for CD31/CD45/CD140a. RNA was extracted from sorted cells and used for RNA sequencing (RNA-seq). **(B)** Top 15 biological processes and KEGG signaling pathways of up- and down-regulated genes from RNA-seq transcriptional profiling of sorted tumor cells versus normal keratinocytes. **(C)** Significantly deregulated keratins. **(D)** Genes significantly altered that cluster in the PI3K-AKT, Jak-Stat, and focal adhesion–ECM receptor interaction KEGG signaling pathways. **(E)** Clustering of biological processes using the MSigDB c5.bp.v6.2 gene set. Red nodes represent up-regulated gene sets and blue nodes represent gene sets down-regulated in the tumor cells. Node size shows the size of gene sets. Nodes that clustered together are classes with same or similar function indication. Lines between the nodes represent association of the gene sets within the nodes. **(F)** Graphical representation of genes/pathways regulated by ALK^F1174L^, based on RNA-seq data.

To dissect the molecular mechanisms underlying the *ALK*^*F1174L*^-driven cSCC in the skin, we isolated tumor epithelial cells (TECs) from *ALK*^*F1174L*^ lesions and normal keratinocytes from their *wild-type* littermates by FACS using a combination of Epcam^+^CD31^−^CD45^−^CD140a^−^ markers as previously described (Nassar et al, 2015) and independent triplicates were subjected to RNA sequencing (Fig 2A). Analysis of differentially expressed keratins revealed a pronounced decrease in the expression level of Krt77 (Krt 1b) and Krt15, indicative of an impaired epithelial differentiation (Fig 2C). On the other hand, several other keratins, including Krt6, Krt7, Krt17, and Krt18 were significantly increased. Krt6, Krt16, and Krt17 are associated with aberrant proliferation, and Krt18 is a marker of embryonal keratinocytes and is often associated with poor differentiation and epithelial-to-mesenchymal transition (EMT) in cSCC (Fig 2C) (Watanabe et al, 1995). Gene ontology (GO) term analysis provided further insights into biological processes enriched in *ALK*^*F1174L*^ TECs. Among significantly up-regulated processes were cell adhesion/migration and proliferation, along with alterations in metabolic processes (Fig 2B and E). The latter overall indicated a shift towards an anabolic and glycolytic (Warburg-like) metabolic phenotype, an additional hallmark that *ALK*^*F1174L*^ cells would gain in support of transformation. In addition, Kyoto Encyclopeida of Genes and Genomes (KEGG) analysis revealed *ALK*^*F1174L*^-associated signature including alterations in several signaling pathways including FAK and ECM, PI3K-AKT, and JAK-STAT3 (Fig 2B and D). Moreover, *ALK*^*F1174L*^ expression resulted in an increase of Mek1 (Map2k1) expression (Fig 2D and F). Interestingly to note is that this signaling signature has been described as characteristic of cSCC development (Ratushny et al, 2012). The expression profile of *ALK*^*F1174L*^-driven tumorigenesis reveals the lack of Sonic Hedgehog (Shh) signaling pathway, which is characteristic of the typical BCC, demonstrating that *ALK*^*F1174*^ expression leads to cSCC formation without any cellular and molecular signs of concomitant BCC development.

**Figure 3.**
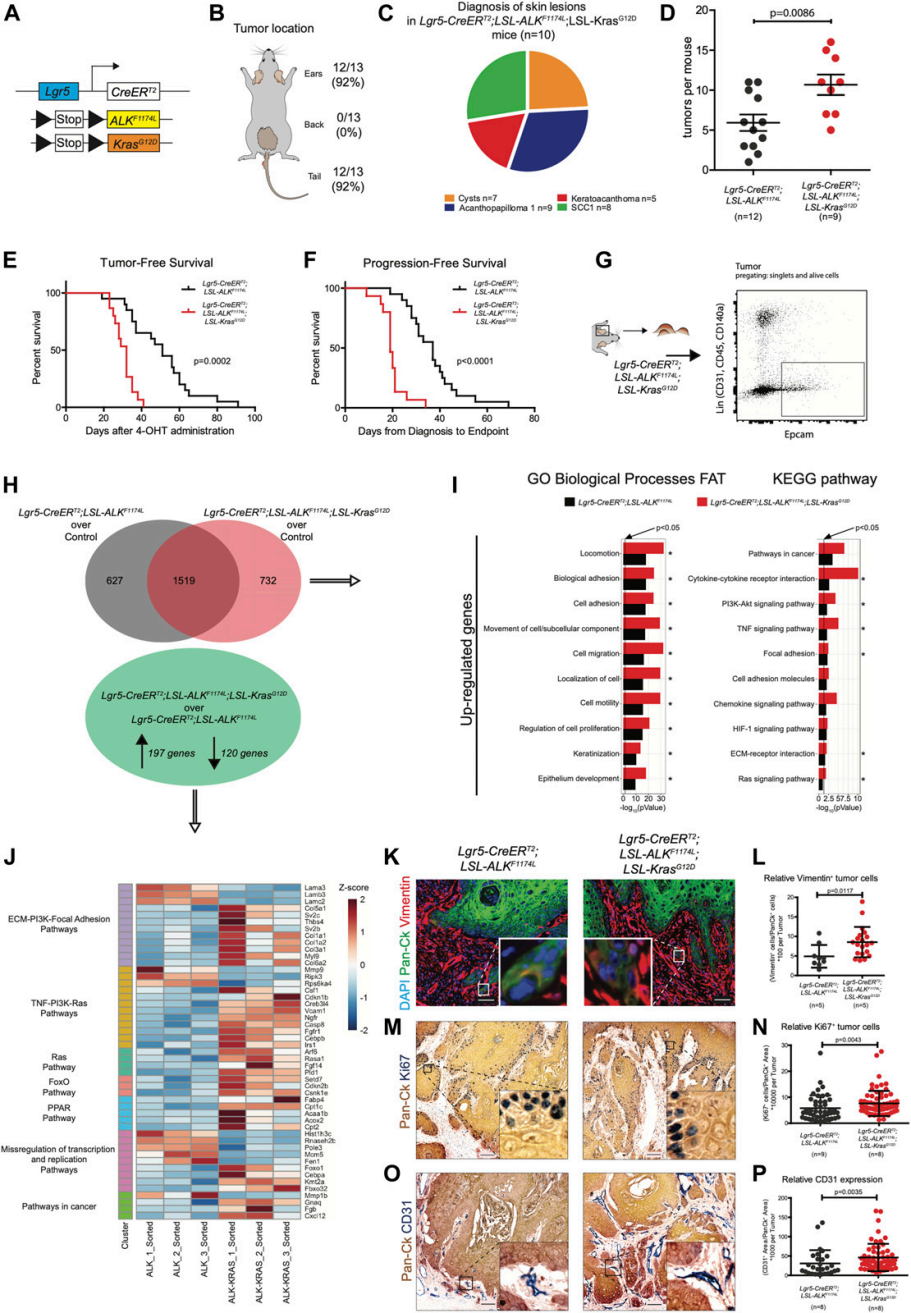
Kras^G12D^ synergizes with ALK^F1174L^ increasing tumorigenicity, epithelial-to-mesenchymal transition properties, and vascularization and proliferation. **(A)** Graphical representation of *Lgr5-CreER*^*T2*^*;LSL-ALK*^*F1174L*^*;LSL-Kras*^*G12D*^ genotype. **(B)** Distribution of tumor formation per location in *Lgr5-CreER*^*T2*^*;LSL-ALK*^*F1174L*^*;LSL-Kras*^*G12D*^ mice. **(C)** Pie chart representing the number of mice that developed the listed skin lesions out of total lesions diagnosed. **(D)** Number of tumors per mouse in *Lgr5-CreER*^*T2*^*;LSL-ALK*^*F1174L*^ and *Lgr5-CreER*^*T2*^*;LSL-ALK*^*F1174L*^*;LSL-Kras*^*G12D*^ mice. Each dot represents the number of tumors per mouse. Mean ± standard error of the mean (5.917 ± 1.026; n = 12; 10.67 ± 1.280 n = 9; two-tailed *t* test *P* = 0.0086). **(E)** Tumor-free survival of *Lgr5-CreER*^*T2*^*;LSL-ALK*^*F1174L*^ (n = 15, median 47 d) and *Lgr5-CreER*^*T2*^*;LSL-ALK*^*F1174L*^*;LSL-Kras*^*G12D*^ (n = 13, median 29 d) mice. Log-rank (Mantel–Cox) test; *P* = 0.0002, HR = 0.1461. **(F)** Progression-free survival of *Lgr5-CreER*^*T2*^*;LSL-ALK*^*F1174L*^ (n = 15, median 37 d) and *Lgr5-CreER*^*T2*^*;LSL-ALK*^*F1174L*^*;LSL-Kras*^*G12D*^ (n = 13, median 19 d) mice. Log-rank (Mantel–Cox) test; *P* < 0.0001, HR = 0.07945. **(G)** Ear tumor from *Lgr5-CreER*^*T2*^*;LSL-ALK*^*F1174L*^*;LSL-Kras*^*G12D*^ mice were prepared as a single-cell suspension, and tumor cells were isolated by FACS for EpCAM expression e-negative selected for CD31/CD45/CD140a. RNA from sorted cells was used for RNA-seq. **(H)** Above: Venn diagram representing deregulated genes in tumor cells over controls. Below: significantly altered genes in *Lgr5-CreER*^*T2*^*;LSL-ALK*^*F1174L*^*;LSL-Kras*^*G12D*^ over *Lgr5-CreER*^*T2*^*;LSL-ALK*^*F1174L*^ tumor cells. **(I)** Significantly altered biological processes and KEGG signaling pathways in *Lgr5-CreER*^*T2*^*;LSL-ALK*^*F1174L*^ and *Lgr5-CreER*^*T2*^*;LSL-ALK*^*F1174L*^*;LSL-Kras*^*G12D*^ tumors. Horizontal columns represent the *P*-value of the analysis of the up-regulated genes from RNA-seq transcriptional profiling of sorted tumor cells versus normal keratinocytes. Processes and pathways marked by * are significantly changed also in the analysis of transcriptomes of *Lgr5-CreER*^*T2*^*;LSL-ALK*^*F1174L*^ versus *Lgr5-CreER*^*T2*^*;LSL-ALK*^*F1174L*^*;LSL-Kras*^*G12D*^ tumor cells. **(J)** Genes significantly regulated from RNA-seq analysis, between *Lgr5-CreER*^*T2*^*;LSL-ALK*^*F1174L*^ and *Lgr5-CreER*^*T2*^*;LSL-ALK*^*F1174L*^*;LSL-Kras*^*G12D*^ tumor cells that cluster in the indicated KEGG signaling pathways. **(K)** Representative immunofluorescences of *Lgr5-CreER*^*T2*^*;LSL-ALK*^*F1174L*^ and *Lgr5-CreER*^*T2*^*;LSL-ALK*^*F1174L*^*;LSL-Kras*^*G12D*^ ear tumors immuno-labelled with DAPI, pan-cytokeratin (Pan-Ck), and vimentin antibodies. **(L)** Cells that co-expressed Pan-Ck and vimentin were counted as cells in epithelial-to-mesenchymal transition. Every tumor arising in the mice was analyzed and relative quantification is represented by a dot. Analysis of tumors from five *Lgr5-CreER*^*T2*^*;LSL-ALK*^*F1174L*^ and five *Lgr5-CreER*^*T2*^*;LSL-ALK*^*F1174L*^*;LSL-Kras*^*G12D*^ mice showed an increased number of relative vimentin^+^ cells in *Lgr5-CreER*^*T2*^*;LSL-ALK*^*F1174L*^*;LSL-Kras*^*G12D*^ tumors (4.907 ± 2.904; 8.547 ± 3.885. Mann–Whitney test *P* = 0.0117). Scale bars = 50 µm. **(M)** Representative immunohistochemistry of *Lgr5-CreER*^*T2*^*;LSL-ALK*^*F1174L*^ and *Lgr5-CreER*^*T2*^*;LSL-ALK*^*F1174L*^*;LSL-Kras*^*G12D*^ ear tumors immuno-labelled with Pan-Ck and with Ki67 antibodies, to identify tumors and proliferating cells. **(N)** Analysis of tumors from nine *Lgr5-CreER*^*T2*^*;LSL-ALK*^*F1174L*^ and eight *Lgr5-CreER*^*T2*^*;LSL-ALK*^*F1174L*^*;LSL-Kras*^*G12D*^ mice showed an increased relative number of proliferating cells in the *Lgr5-CreER*^*T2*^*;LSL-ALK*^*F1174L*^*;LSL-Kras*^*G12D*^ tumors (5.786 ± 4.958; 7.614 ± 4.828. Mann–Whitney test *P* = 0.0043). Scale bars = 100 µm. **(O)** Representative immunohistochemistry of *Lgr5-CreER*^*T2*^*;LSL-ALK*^*F1174L*^ and *Lgr5-CreER*^*T2*^*;LSL-ALK*^*F1174L*^*;LSL-Kras*^*G12D*^ ear tumors immuno-labelled with Pan-Ck and with CD31 antibodies, to identify tumors and vessels. **(P)** Every tumor arising in the mice was analyzed and relative quantification is represented by a dot. Analysis of tumors from eight *Lgr5-CreER*^*T2*^*;LSL-ALK*^*F1174L*^ and eight *Lgr5-CreER*^*T2*^*;LSL-ALK*^*F1174L*^*;LSL-Kras*^*G12D*^ mice showed an increased relative area occupied by vessels within the *Lgr5-CreER*^*T2*^*;LSL-ALK*^*F1174L*^*;LSL-Kras*^*G12D*^ tumors (30.75 ± 34.34; 46.10 ± 35.24. Mann–Whitney test *P* = 0.0035). Scale bars = 100 µm. **(J, K, L)** Data showed as mean ± SD.

Similarly to the DMBA/TPA model of mouse carcinogenesis, human SCCs are often associated with mutations in the *RAS* genes (van der Schroeff et al, 1990; Spencer et al, 1995). Overexpression of either *Hras* or *Kras* in mouse skin initiates tumorigenesis (Brown et al, 1998; Vitale-Cross et al, 2004; Caulin et al, 2007). The analysis of RNA-seq data obtained from the comparison of *WT* ear keratinocytes and *ALK*^*F1174L*^ TECs did not reveal any significant deregulation in the *Ras* signature. Moreover, our data on the co-occurrence of mutations in *RAS* and *ALK* genes in human patients did not reveal any strong evidence suggesting that ALK and RAS are mutually exclusive drivers of cSCC (Fig S2E). Thus, we sought to functionally determine whether *ALK*^*F1174L*^ can cooperate with *Kras* in driving SCC development. To answer this question, we have crossed *ALK*^*F1174L*^ *Lgr5-CreERT2* mice with *Kras*^*G12D*^ strain (Fig 3A) (Jackson et al, 2001). Within less than 40 d, all mice developed ulcerative lesions on the ears and tails (n = 13) (Fig 3B and E). As shown in Fig 3B, the tumor penetrance/incidence remained as high as in the *ALK*^*F1174L*^ transgene and reached 92% in ear and tail areas. However, similarly to *ALK*^*F1174L*^ overexpression, no tumors were seen in the back skin of *ALK*^*F1174L*^*Kras*^*G12D*^ *Lgr5-CreERT2* mice. In analogy to *ALK*^*F1174L*^ *Lgr5-CreERT2* mice, the histological analysis revealed the presence of four distinct histological entities including, cysts, AP, KA, and SCC1 (Figs 3C and S3C–E). The addition of *Kras*^*G12D*^ transgene resulted in an increased number of tumors per mouse as compared with *ALK*^*F1174L*^ *Lgr5-CreERT2* mice (Fig 3D). Moreover, *ALK*^*F1174L*^*Kras*^*G12D*^ *Lgr5-CreERT2* mice showed signs of aggressive SCC (size, ulceration, and bleeding) at earlier time points as compared with *ALK*^*F1174L*^ *Lgr5-CreERT2* mice (median 19 d post induction versus 37, d respectively, *P* < 0.0001) (Fig 3F). Taken together, these data strongly suggest that *Kras*^*G12D*^ oncogene cooperates with *ALK*^*F1174L*^ in driving the pathogenesis of cSCC.

To determine the molecular mechanisms underlying this cooperative effect, we have isolated TECs from tumors of *ALK*^*F1174L*^ *Lgr5-CreERT2* and *ALK*^*F1174L*^*Kras*^*G12D*^ *Lgr5-CreERT2* (Fig 3G) using previously described FACS protocol (Nassar et al, 2015) and performed RNA sequencing. GO and KEGG analysis highlighted several major alterations upon the addition of *Kras*^*G12D*^ oncogene (Fig 3H–J). Among these, cell adhesion/migration and ECM remodeling were suggestive of an EMT process initiated upon *Kras*^*G12D*^. To further test it, we performed immunostaining for vimentin, a mesenchymal marker, and quantified the number of cells double positive for pan-CK and vimentin (Fig 3K). As shown in Fig 3L, double transgenic *ALK*^*F1174L*^*Kras*^*G12D*^ *Lgr5-CreERT2* mice showed higher number of pan-Ck^+^/Vim^+^ cells as than single *ALK*^*F1174L*^ transgene (n = 5; 5, *P* = 0.0117, mean ± SD: 4.907 ± 2.904; 8.547 ± 3.885). Furthermore, based on our RNA-seq data, double transgenic TECs displayed an increased proliferation. To verify this, we performed immunostaining for Ki67, a marker of cell proliferation and quantified number of Pan-Ck^+^/Ki67^+^ cells within tumors (Fig 3M and N). Indeed, in alignment with RNA-seq data, we show that *Kras*^*G12D*^ oncogenic induction resulted in a pronounced increase in the proliferative index of cancer cells. In addition, among the most significantly regulated keratins, we identified Krt4, Krt8, and Krt19 strongly up-regulated in *ALK*^*F1174L*^*Kras*^*G12D*^ TECs (Fig S3F). Krt4, Krt8, and Krt19 were previously described as keratins associated with stem cell potential, dedifferentiation, proliferation, and invasion (Watanabe et al, 1995). To examine the degree of vascularization within individual tumors, we used a well-defined marker of angiogenesis, CD31, and quantified its relative expression (Fig 3O and P). Our results revealed that *Kras*^*G12D*^ significantly increased the expression of CD31, suggesting the presence of enhanced vascularization in tumors of *ALK*^*F1174L*^*Kras*^*G12D*^ *Lgr5-CreERT2* mice. Thus, the cooperation between *ALK*^*F1174*^ and *Kras*^*G12D*^, leading to the more aggressive type of cSCC, manifests through a combination of cellular mechanisms, including induction of EMT, increase in, proliferation and enhanced vascularization.

**Figure S4.**
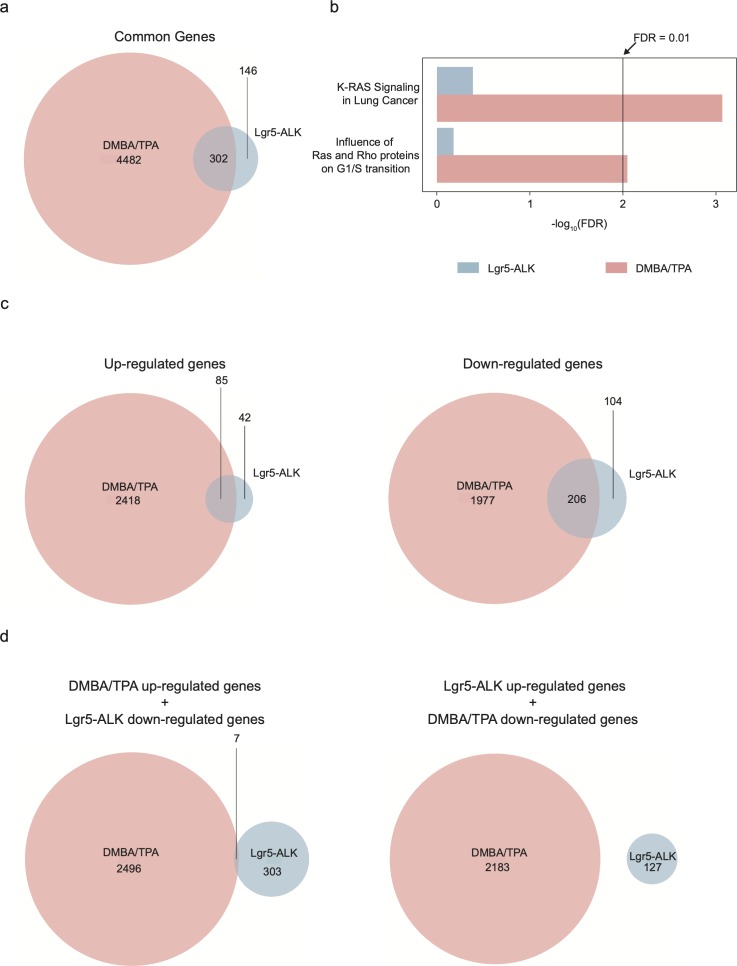
**Comparison of the signaling pathways in DMBA/TPA-induced cSCC and *ALK^F1174L^*-induced cSCC. (A)** Common genes between *Lgr5-CreER*^*T2*^*;LSL-ALK*^*F1174L*^ tumor epithelial cells and DMBA/TPA genetic signatures. **(B)** Analysis of Ras-associated pathways in both mouse models reveals that genes from these pathways are significantly altered in DMBA/TPA and not in Lgr5-ALK mouse model. **(C)** Number of significantly up- or down-regulated genes in both mouse models. Overlapping genes can indicate common genes involved in carcinogenesis of cSCC. **(D)** Number of genes significantly up-regulated in one model versus down-regulated in the other model. Non-overlapping genes can indicate model-dependent genes involved in the carcinogenesis of cSCC.

**Figure S5.**
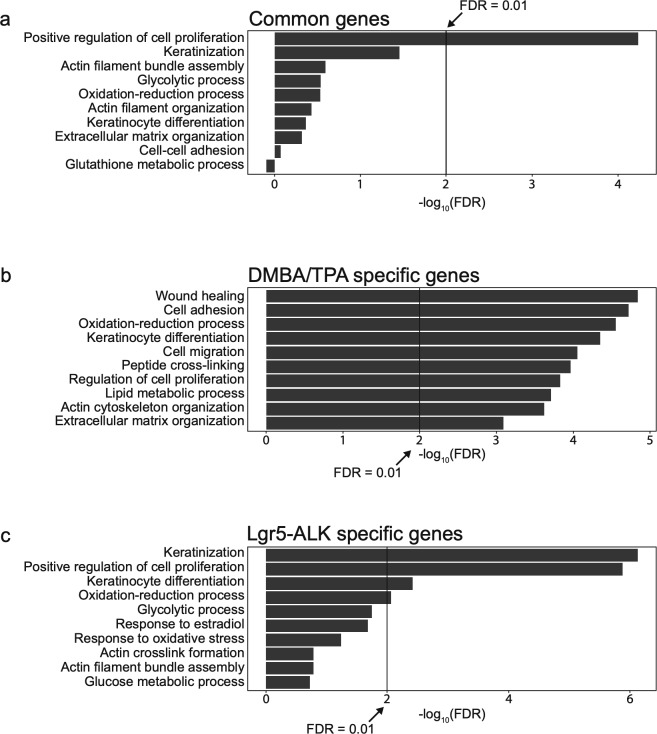
**Characterization of the specific biological process in DMBA/TPA-induced cSCC and *ALK^F1174L^*-induced cSCC. (A)** Top 10 biological processes associated with different group of genes and pathways associated with common genes significantly up- and down-regulated in both mouse models. **(B)** Pathways associated with genes significantly up- and down-regulated specifically in DMBA/TPA mouse model. **(C)** Pathways associated with genes significantly up- and down-regulated specifically in *Lgr5-CreER*^*T2*^*;LSL-ALK*^*F1174L*^ mouse model.

To delve further into the question of whether DMBA/TPA tumors exhibit molecular signature similar or distinct of the one observed in ALK-driven cSCC, we compared genetic profiles of *ALK*^*F1174L*^ TECs to the previously published signature of DMBA/TPA tumors (Nassar et al, 2015) (Figs S4 and S5). 302 overlapping genes emerged as a result of this comparison, including 85 commonly up-regulated and 206 down-regulated (Fig S4A and C). The analysis of Ras-associated pathways in both mouse models confirms our previous observations and reveals that ALK executes a specific pro-tumorigenic program that is distinct of Ras-driven cSCC (Fig S4B and D).

**Figure 4.**
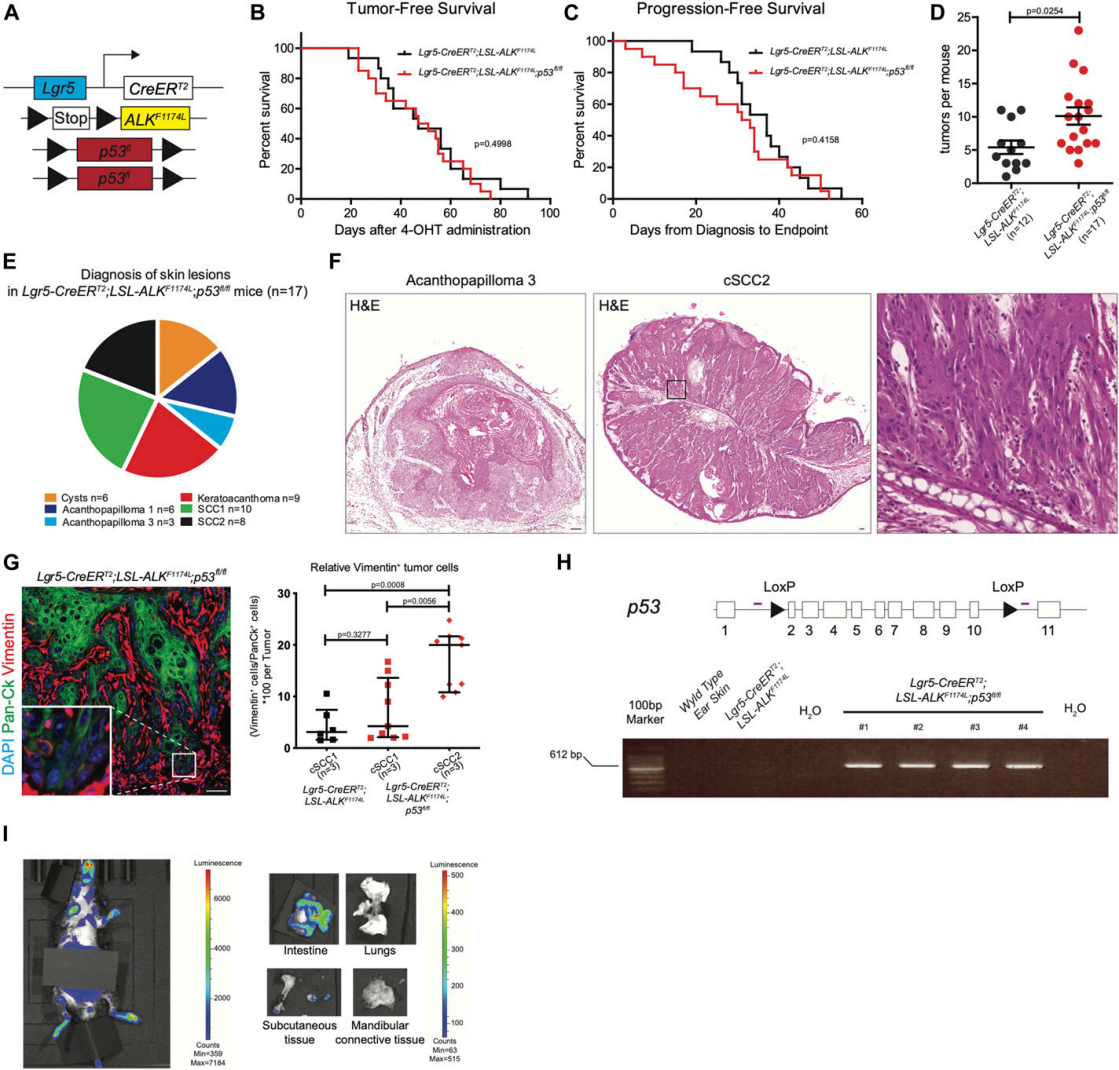
p53 conditional KO increased tumorigenicity driven by ALK^F1174L^. **(A)** Graphical representation of *Lgr5-CreER*^*T2*^*;LSL-ALK*^*F1174L*^*;p53*^*fl/fl*^ genotype. **(B)** Tumor-free survival of *Lgr5-CreER*^*T2*^*;LSL-ALK*^*F1174L*^ (n = 15, median 47 d) and *Lgr5-CreER*^*T2*^*;LSL-ALK*^*F1174L*^*;p53*^*fl/fl*^ (n = 20, median 49 d) mice. Log-rank (Mantel–Cox) test; *P* = 0.4998, HR = 0.7834. **(C)** Progression-free survival of *Lgr5-CreER*^*T2*^*;LSL-ALK*^*F1174L*^ (n = 15, median 37 d) and *Lgr5-CreER*^*T2*^*;LSL-ALK*^*F1174L*^*;p53*^*fl/fl*^ (n = 20, median 32 d) mice. Log-rank (Mantel–Cox) test; *P* = 0.4158, HR = 0.7478. **(D)** Number of tumors per mouse in *Lgr5-CreER*^*T2*^*;LSL-ALK*^*F1174L*^ and *Lgr5-CreER*^*T2*^*;LSL-ALK*^*F1174L*^*;p53*^*fl/fl*^ mice. Each dot represents the number of tumors in a specific mouse. Mean ± standard error of the mean (5.917 ± 1.026; n = 12; 10.12 ± 1.3 n = 17; Two-tailed *t* test *P* = 0.0254). **(E)** Pie chart representing the number of mice that developed the listed skin lesions out of total lesions diagnosed. **(F)** Representative hematoxylin and eosin (H&E) staining of acanthopapilloma3 and multiple SCC2 from ear skin of *Lgr5-CreER*^*T2*^*;LSL-ALK*^*F1174L*^*;p53*^*fl/fl*^ mice. In the magnification, it is possible to appreciate mesenchymal-like features of tumor cells and pronounced nuclear atypia. Scale bars = 100 µm. **(G)** Representative immunofluorescences of *Lgr5-CreER*^*T2*^*;LSL-ALK*^*F1174L*^*;p53*^*fl/fl*^ ear tumor immuno-labelled with DAPI, pan-cytokeratin (Pan-Ck), and vimentin antibodies. Scale bar = 50 µm. Cells that co-expressed Pan-Ck and vimentin were counted as cells in epithelial-to-mesenchymal transition (EMT). All tumors diagnosed as cSCC type 1 or 2 from different mice were analyzed, and quantification of the relative number of cells in EMT per tumor is represented by a dot. Analysis of tumors showed that no significant changes were observed between SCC1 tumors of three *Lgr5-CreER*^*T2*^*;LSL-ALK*^*F1174L*^ or three *Lgr5-CreER*^*T2*^*;LSL-ALK*^*F1174L*^*;p53*^*fl/fl*^ mice (4.380 ± 1.425; 7.360 ± 1.996. Mann–Whitney test *P* = 0.3277). Strong increase in the EMT rate was instead noted in SCC2 tumors when compared with SCC1 tumors of the same cohort of mice (7.360 ± 1.996; 17.10 ± 1.875. Mann–Whitney test *P* = 0.0056) or to Lgr5-CreERT2;LSL-ALKF1174L mice (4.380 ± 1.425; 17.10 ± 1.875. Mann–Whitney test *P* = 0.0008). All values are showed as median with 95% confidence interval. **(H)** Above: the schematic structure of the *p53* floxed allele and graphical representation of PCR strategy to determine the recombination efficiency of LoxP sites that drives the removal of exons 2–10 of the *p53* gene. White boxes represent exons, arrowheads represent the LoxP sites, and purple lines indicate primers position. Below: PCR analysis of recombination in different tissues. The 612-bp band present in the *Lgr5-CreER*^*T2*^*;LSL-ALK*^*F1174L*^*;p53*^*fl/fl*^ tumors confirms that recombination occurred. **(I)** Representative pictures of in vivo imaging system (IVIS). Analysis performed to detect metastasis in vivo (left) and ex vivo (right). The grey box is used to cover a strong luciferase signal in the gut. The strong luciferase expression observed in the intestinal epithelium of the gut is likely due to the residual amount of 4-OHT entering systemic circulation (mice transferring 4-OHT by scratching their ears and subsequently licking their paws).

**Figure S6.**
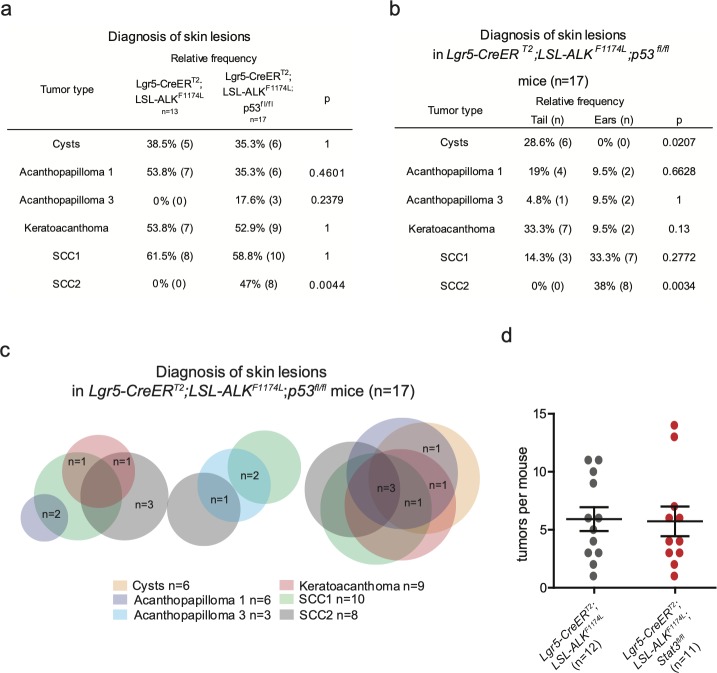
**Histopathological characterization of skin lesions observed in *LSL-ALK^F1174L ^p53^fl/fl^ Lgr5-CreERT2*. (A, B, C)** Evaluation of the skin lesions of *Lgr5-CreER*^*T2*^*;LSL-ALK*^*F1174L*^*;p53*^*fl/fl*^ mice. **(A)** The *P*-value has been calculated by Fisher’s exact test in contingency table comparing the proportion of *Lgr5-CreER*^*T2*^*;LSL-ALK*^*F1174L*^ and *Lgr5-CreER*^*T2*^*;LSL-ALK*^*F1174L*^*; p53*^*fl/fl*^ mice that developed the corresponding skin lesion out of total mice per group. **(B)** The *P*-value has been calculated by Fisher’s exact test in contingency table comparing the proportion of the corresponding skin lesion out of the total lesions per location (tail or ear). **(C)** Venn diagram representing the distribution of the different skin lesions in the mice. **(D)** Number of tumors per mouse in *Lgr5-CreER*^*T2*^*;LSL-ALK*^*F1174L*^ and *Lgr5-CreER*^*T2*^*;LSL-ALK*^*F1174L*^*;Stat3*^*fl/fl*^ mice. Each dot represents the number of tumors in a specific mouse. Mean ± standard error of the mean (5.917 ± 1.026 n = 12, 5.727 ± 1.280 n = 11; Two-tailed *t* test *P* = 0.9084).

Functional loss of *TP53* gene is a hallmark of human SCC (van Kranen et al, 1995), and loss of *p53* gene combined with *Kras*^*G12D*^ overexpression promotes invasive SCC in mice (Lapouge et al, 2011; White et al, 2011). Our analysis of human cSCC patients (Fig S2E) revealed the co-occurrence between *ALK* mutations and mutations in *p53* gene (68 cases analyzed, 0 cases displayed ALK only mutations, and 19 cases displayed mutations in both genes). To determine whether the loss of *p53* can promote metastases in *ALK*^*F1174*^-driven model of SCC, we have crossed *ALK*^*F1174L*^ *Lgr5-CreERT2* mice to *p53*^*fl/fl*^ allele (Marino et al., 2000) (Fig 4A). Neither tumor-free survival nor progression-free survival were significantly altered upon *p53* loss (Fig 4B and C). However, the frequency of tumors per mouse was increased in *p53*^*fl/fl*^ as compared with *ALK*^*F1174*^ single transgene (Fig 4D). Histological examination revealed several *p53* loss–associated alterations. In addition to a higher degree of nuclear atypia, tumors isolated from *ALK*^*F1174L*^*p53*^*fl/fl*^ *Lgr5-CreERT2* mice contained not only cysts, KA, AP, and SCC1 but also included more aggressive histological subtypes, AP3 and SCC2 (Figs 4E and F and S6A–C). Because *p53* loss leads to an invasive cSCC in several mouse models of cSCC, we decided to investigate whether tumors obtained from *ALK*^*F1174L*^*p53*^*fl/fl*^ *Lgr5-CreERT2* mice were characterized by a change in the EMT signature. We have decided to evaluate the expression of vimentin, a protein which is not only expressed at low levels in the dermal cells of the skin but is also one of markers of cells undergoing EMT (White et al, 2011). The quantification of the relative number of Vim^+^/Pan-Ck^+^ cells within these tumors highlighted the presence of a greater number of cells with invasive characteristics, particularly in SCC2 (Fig 4G). The recombination in *p53* floxed allele was verified using PCR analysis as shown in Fig 4H (Marino et al., 2000). In summary, these data indicate that the *p53* loss resulted in the most aggressive histological subtype of *ALK*^*F1174L*^-driven primary SCC. Our data are in agreement with several other reports demonstrating that *Kras*^*G12D*^ when combined with the loss of *p53* gene results in a spindle cell, high-grade SCC (Lapouge et al, 2011; White et al, 2011, 2014; White & Lowry, 2011).

To monitor metastases, we took the advantage of the presence of *luc* reporter gene and subjected *ALK*^*F1174L*^*p53*^*fl/fl*^ *Lgr5-CreERT2* mice to an in vivo live imaging at the time point when the termination criteria were met and before euthanizing (n = 7). As shown in Fig 4I, luciferase expression was readily detected in the skin as well as in the gut of *ALK*^*F1174L*^*p53*^*fl/fl*^ *Lgr5-CreERT2* mice. However, no signs of distant metastases were detected in the internal organs (Fig 4I). This could be explained either by strict termination criteria of our study or low sensitivity of IVIS imaging, and therefore, we cannot completely rule out the occurrence of micrometastases with parameters under our instrumental detection limits.

Among down-regulated genes upon *ALK*^*F1174L*^ overexpression, we have identified that several inhibitors of signal transducer and activator of transcription 3 (Stat3) were altered. We have observed down-regulation of the protein inhibitor of activated STAT, Pias3, a specific inhibitor of Stat3, which prevents DNA-binding activity of Stat3 and thereby abolished Stat3-mediated transcription (Chung et al, 1997). Moreover, the expression of another inhibitor of Stat3, Socs7 (Martens et al, 2005), was also decreased (Fig 2D). In addition, the expression of the tyrosine phosphatase Ptpn6 (Shp1), which was shown to dephosphorylate Stat3 but not Stat1 or Stat5 (Demosthenous et al, 2015), was also changed based on our RNA-seq results. Previous reports showed that the expression of constitutively active form of Stat3 driven by the K5 promoter (K5.Stat3C mice) resulted in an increased number of tumors as well as a shorter latency period after DMBA/TPA treatment (Chan et al, 2008) and Stat3-deficient mice are resistant to chemically induced carcinogenesis (Chan et al, 2004).

**Figure 5.**
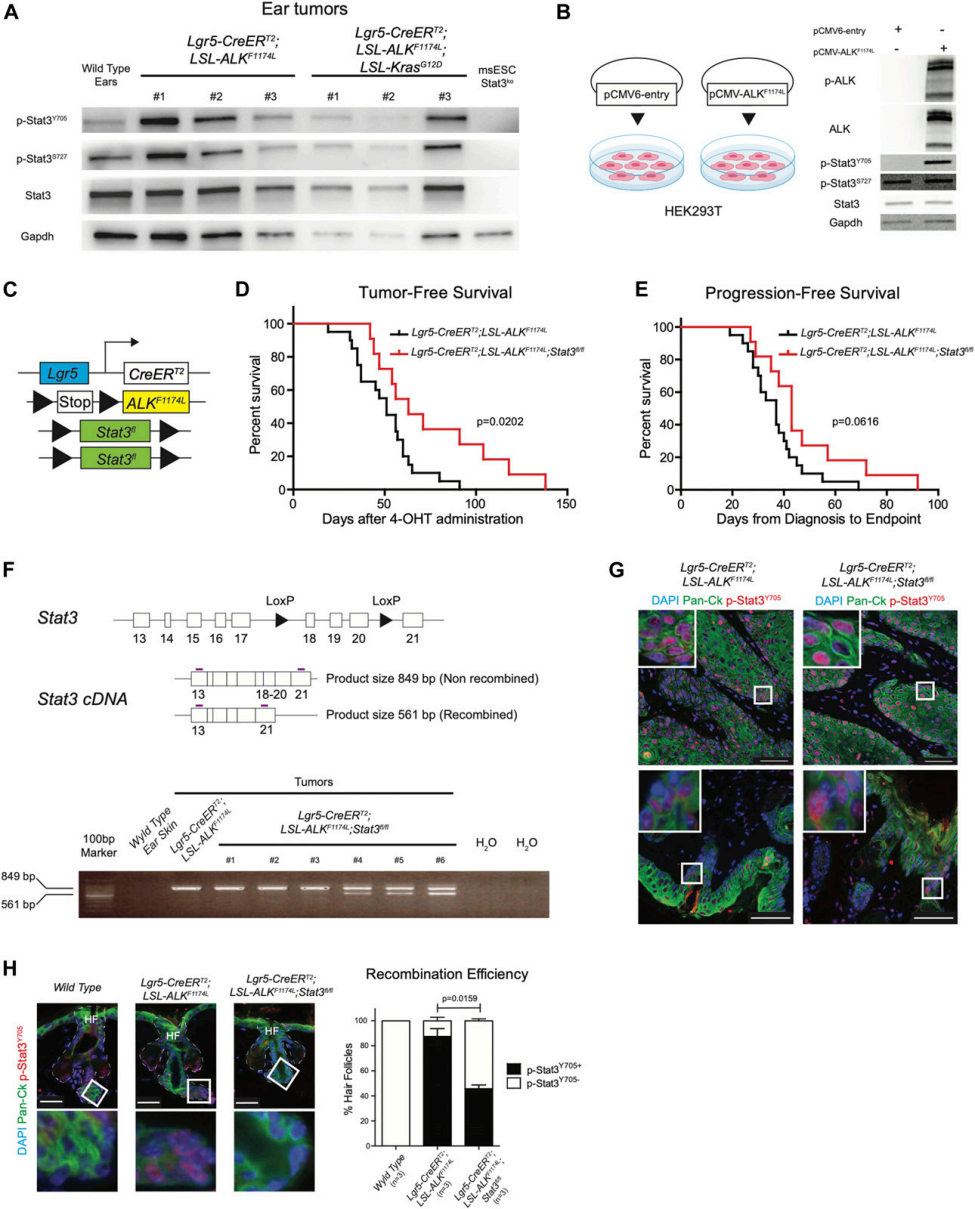
Stat3 is indispensable for ALK^F1174L^-driven tumorigenicity. **(A, B)** Western blot analysis of phosphorylation status of Stat3 in (A) *Lgr5-CreER*^*T2*^*;LSL-ALK*^*F1174L*^ and *Lgr5-CreER*^*T2*^*;LSL-ALK*^*F1174L*^*;LSL-Kras*^*G12D*^ tumors and in (B) HEK293T cell line transiently expressing the *ALK*^*F1174L*^ transcript or control. **(C)** Graphical representation of *Lgr5-CreER*^*T2*^*;LSL-ALK*^*F1174L*^*;Stat3*^*fl/fl*^ genotype. **(D)** Tumor-free survival of *Lgr5-CreER*^*T2*^*;LSL-ALK*^*F1174L*^ (n = 20, median 51 d) and *Lgr5-CreER*^*T2*^*;LSL-ALK*^*F1174L*^*;Stat3*^*fl/fl*^ (n = 11, median 63 d) mice. Log-rank (Mantel–Cox) test; *P* = 0.0202, HR = 2.540. **(E)** Progression-free survival of *Lgr5-CreER*^*T2*^*;LSL-ALK*^*F1174L*^ (n = 20, median 37 d) and *Lgr5-CreER*^*T2*^*;LSL-ALK*^*F1174L*^*;Stat3*^*fl/fl*^ mice (n = 11, median 43 d). Log-rank (Mantel–Cox) test; *P* = 0.0616, HR = 2.066. **(F)** Above: structure of the *Stat3* floxed allele and graphical representation of PCR strategy to analyze recombination efficiency. Recombined allele will produce a shorter mRNA missing exons 18-19-20. White boxes represent exons, arrowheads represent the LoxP sites, and purple lines represent the primers. Below: RT-PCR analysis of different tissues. Whereas *wild-type* ear skin shows no detectable expression of *Stat3*, only 3/6 of the *Lgr5-CreER*^*T2*^*;LSL-ALK*^*F1174L*^*;Stat3*^*fl/fl*^ tumors analyzed show a mixed expression of full-length and truncated *Stat3*. Number of tumors per mouse in *Lgr5-CreER*^*T2*^*;LSL-ALK*^*F1174L*^ and *Lgr5-CreER*^*T2*^*;LSL-ALK*^*F1174L*^*;Stat3*^*fl/fl*^ mice. Mean ± standard error of the mean (5.917 ± 1.026 n = 12, 5.727 ± 1.280 n = 11; two-tailed *t* test *P* = 0.9084). **(G)** Representative immunofluorescence images of *Lgr5-CreER*^*T2*^*;LSL-ALK*^*F1174L*^, *Lgr5-CreER*^*T2*^*;LSL-ALK*^*F1174L*^*;Stat3*^*fl/fl*^ ear tumors immuno-labelled with DAPI, pan-cytokeratin (Pan-Ck), and p-Stat3^Y705^ antibodies show phosphorylation of Stat3 within the tumors (above) and in the hyperplastic skin and in the hair follicles (HF) adjacent to the tumor masses (below). To note, all *Lgr5-CreER*^*T2*^*;LSL-ALK*^*F1174L*^*;Stat3*^*fl/fl*^ tumors analyzed (n = 8) showed p-Stat3^Y705^ expression. Scale bars = 50 µm. **(H)** From left to right, *wild-type* ears HFs, and non-hyperplastic *Lgr5-CreER*^*T2*^*;LSL-ALK*^*F1174L*^ and *Lgr5-CreER*^*T2*^*;LSL-ALK*^*F1174L*^*;Stat3*^*fl/fl*^ ear HFs adjacent to the tumor masses have been immuno-labelled with DAPI, pan-cytokeratin (Pan-Ck), and p-Stat3^Y705^ antibodies. Insets display strong p-Stat3^Y705^ expression within the HFs of *Lgr5-CreER*^*T2*^*;LSL-ALK*^*F1174L*^ mice, whereas *wild-type* skin is devoid of p-Stat3^Y705^ expression. Scale bars = 25 µm. Quantification of the recombination was measured as relative number of HFs expressing or not expressing p-Stat3^Y705^. Only non-hyperplastic HFs were being considered for the analysis.

Based on these observations, we hypothesized that expression of *ALK*^*F1174L*^ may trigger Stat3. To test this, we analyzed the protein homogenates from several *ALK*^*F1174L*^ and *ALK*^*F1174L*^*Kras*^*G12D*^ tumors with anti-Y705-Stat3 and anti-S727-Stat3 antibodies using Western blot (Fig 5A). The vast majority of tumors showed phosphorylation of Stat3, whereas Stat3 KO ES cells were devoid of any signal. To evaluate whether *ALK*^*F1174L*^ overexpression can lead to phosphorylation of Stat3, we compared HEK293T cells transiently overexpressing the mutated ALK (*pCMV-ALK*^*F1174L*^) with their corresponding mock control (*pCMV6-entry*). As shown in Fig 5B, the overexpression of *ALK*^*F1174L*^ in HEK293T cells resulted in Stat3 phosphorylation. On the basis of these results, we reasoned that Stat3 might play an essential role in mediating *ALK*^*F1174L*^-driven SCC formation. To test this possibility, we have crossed *ALK*^*F1174L*^ *Lgr5-CreERT2* mice to conditional Stat3 knockout mice (Jacoby et al, 2003) (Fig 5C). *ALK*^*F1174L*^ *Stat3*^*fl/fl*^ *Lgr5-CreERT2* showed prolonged tumor-free survival and progression-free survival as compared with *ALK*^*F1174L*^ *Lgr5-CreERT2* mice. However, tumor formation still occurred (Fig 5D and E). We verified the presence of the recombined *Stat3* floxed allele using PCR strategy (Moh et al, 2007) (Fig 5F). The resulting data revealed that 3/6 analyzed tumors from *ALK*^*F1174L*^ *Stat3*^*fl/fl*^ *Lgr5-CreERT2* mice were partially recombined and 3/6 tumors revealed the lack of the *Stat3* recombined allele (Fig 5F), suggesting that the recombination was partial. To test whether the resulting tumors were still expressing Stat3, we have performed immunostaining for p-Stat3 and observed that 100% of tumors still contained Stat3-positive cells (Fig 5G). This observation is in alignment with the data obtained from the analysis of the recombination efficiency (Fig 5F). We next evaluated the percentage of the recombination and counted the number of HF either positive or negative for p-Stat3 protein (all *WT* HF were devoid of p-Stat3). Whereas the vast majority of HFs in *ALK*^*F1174L*^ *Lgr5-CreERT2* mice showed readily detectable p-Stat3 protein, less than 50% of HF were positive for p-Stat3 in *ALK*^*F1174L*^ *Stat3*^*fl/fl*^ *Lgr5-CreERT2* mice (Figs 5H and S6D). It is plausible that these p-Stat3–positive HFs occurred because of the low recombination efficiency and likely give rise to the tumors observed.

Although our data suggest that *ALK*^*F1174L*^-mediated tumorigenesis, at least in part, might occur via its downstream effector STAT3, further experimental evidence is required to determine the significance of these findings.

**Figure 6.**
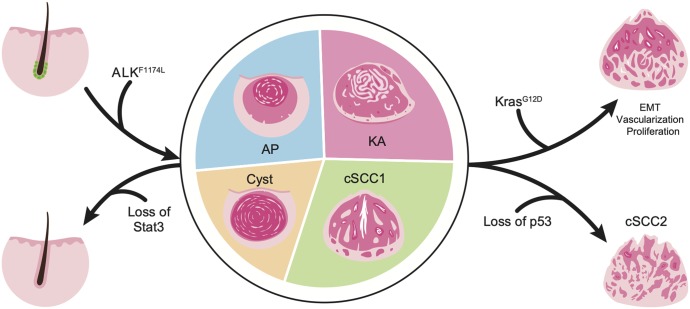
A schematic illustration summarizing main findings. **(A)** Expression of *ALK*^*F1174L*^ in Lgr5^+^ hair follicle stem cells results in formation of different skin lesions as Cysts, acanthopapilloma (AP), keratoacanthoma (KA), or squamous cell carcinoma type 1 (SCC1). Additional *Kras*^*G12D*^ expression leads to increased epithelial to mesenchymal transition, proliferation, and vascularization. Loss of p53 primes to formation of a more aggressive tumor: squamous cell carcinoma type 2 (SCC2). Finally, loss of Stat3 prevents the tumor formation ALK^F1174L^-induced.

Our findings reveal a previously unknown role of oncogenic ALK signaling in cSCC. We show that the expression of a constitutively active *ALK*^*F1174L*^ in mice can lead to the development of aggressive forms of cSCC (Fig 6). On the molecular level, *ALK*^*F1174L*^ can cooperate with known cSCC drivers, including *Kras*^*G12D*^ as well with the loss of tumor suppressor p53. Moreover, our data demonstrate that Stat3 is essential for mediating the oncogenic effect of *ALK*^*F1174L*^. In addition to our data uncovering the essential role of oncogenic *ALK*^*F1174L*^ in mouse cSCC, we show that human samples of cSCC contain *ALK* mutations as well. Therefore, our data provide a rational for oncogenic ALK as a novel therapeutic target and can serve as a basis for the design of future clinical trials.

## Materials and Methods

### Transgenic mice

All animal experiments have been approved by the cantonal veterinary authorities of Zurich, in accordance with Swiss laws. For DNA isolation, mouse biopsies were lysed using lysis buffer (5M NaCl, 2M Tris, pH 88.5, 0.5M EDTA, and 20% SDS) and proteinase K. DNA was precipitated in 50% lysate and 50% isopropanol and centrifuged at 20.817*g* for 30 min. After washing in 70% ethanol, 2 µl of DNA were mixed with 10 µl KAPA Taq ReadyMix with dye (KK1024; Kapa Biosystems) and with 0.8 µl of each primer (10 µM) and finally brought to 20 µl with MilliPore water. The following primers with according melting temperatures were used: 60°C, LSL-ALK_7083_Fw (CCATCAGTGACCTGAAGGAGG) LSL-ALK_7083_Rv (CACGTGCAGAAGGTCCAGC), 60°C, Cre_Fw (CTATCCAGCAACATTTGGGCCAGC) Cre_Rv (CCAGGTTACGGATATAGTTCATGAC), 60°C, LSL-KRAS_Fw1 (TGTCTTTCCCCAGCACAGT) LSL-KRAS_Rv (CTGCATAGTACGCTATACCCTGT) KRAS_Fw2 (GCAGGTCGAGGGACCTAATA), 55°C, Stat3_floxed_Fw (TTGACCTGTGCTCCTACAAAAA) Stat3_floxed_Rv (CCCTAGATTAGGCCAGCACA), 60°C, p53-oIMR8543_Fw (GGTTAAACCCAGCTTGACCA) p53-oIMR8544_Rv (GGAGGCAGAGACAGTTGGAG), 58°C, iCre-K5_Fw (CTCTGACAGATGCCAGGACA) iCre-K5_Rv (TCTCTGCCCAGAGTCATCCT), 60°C, K14-CreErt2_T_Fw (CGCATCCCTTTCCAATTTAC) K14-CreErt2_T_Rv (GGGTCCATGGTGATACAAGG) K14-CreErt2_C_Fw (CTAGGCCACAGAATTGAAAGATCT) K14-CreErt2_C_Rv (GTAGGTGGAAATTCTAGCATCATCC) PCRs were run on a Thermocycler (T300; Biometra) using the following program. 5 min at 95°C, 35 times: denaturation for 40 min at 95°C, annealing for 40 min at MT, 40 min of elongation at 72°C, and final elongation for 10 min at 72°C. The PCR products were run on 2% agarose (in Tris-acetate-EDTA [TAE]) gels containing RedSafe (21141; JH Science) at 90V until bands were nicely separated and subsequently visualized using BioDoc-It Imaging System (Ultra-Violet Products). All transgenic mice used for experiments were maintained in a C57Bl6/129-mixed background. Lgr5tm1(cre/ERT2)Cle (MGI ID: 3764660), Stat3tm2Aki (MGI ID: 1926816), and Tg(KRT14-cre/ERT)20Efu (MGI ID: 2446606) were purchased from The Jackson Laboratory. Tg(Th-ALK*F1174L)2Loch mice were created in the Laboratory of Prof. JH Schulte (Berlin, Germany). Tg(KRT5-cre/PGR)1Der (MGI ID: 2651408) mice were kindly obtained from Laboratory of Prof. M Van Der Broek (Zurich, Switzerland). Tg(Krt1-15-cre/PGR)22Cot mice were generously got from Laboratory of Prof. Y Barrandon (Lausanne, Switzerland). Trp53tm1Brn (MGI ID: 1931011) and B6.129S4-Krastm4Tyj (MGI ID: 2429948) mice were donated by Laboratory of TR Graft (Zurich, Switzerland). The recombination efficiency PCRs were performed as previously described for p53 (Marino et al., 2000) and Stat3 (Moh et al, 2007).

### Induction of Cre activity, termination criteria, and in vivo imaging system

For activation of Cre activity, 6–12-wk-old mice were shaved on the back skin and 4-Hydroxytamoxifen (4-OHT) 25 mg/ml (≥70% Z isomer H7904; Sigma-Aldrich) was topically administered for three consecutive days, using a small paint brush on both ears (inside and outside), on the tail and on the shaved patch of the back skin. The mice were euthanized when termination criteria was reached. In most cases, size of tumors (>0.5 cm of diameter, n = 2) was the adopted termination criteria. Secondary, mice were euthanized if developed one tumor bigger than 1 cm of diameter or if tumor was ulcerative or bleeding. Mice that showed wounds (signs of fights) were withdrawn from experiment. On the day of euthanizing, the animals were anesthetized with isoflurane (B506; Abbott) and shaved with a waxing cream (Veet). Then mice have been injected with 150 mg/kg body weight of XenoLight D-Luciferin-K+ Salt Bioluminescent Substrate (122799; PerkinElmer) and imaged with IVIS Lumina III (PerkinElmer). In vivo and ex vivo analysis was performed, and tissue was collected according with the signal acquired for histological analysis. Skin, organs, and tumors were embedded in paraffin for immunohistochemistry and hematoxylin and eosin staining.

### Immunofluorescence and immunohistochemistry and hematoxylin & eosin (H&E)

Mouse skin and tumor samples were fixed in Roti-Histofix (P078.3; Roth) for 2 h. Then washed in PBS and embedded in paraffin (MEDITE PURE Paraffin, 40-0020-00; MEDITE). Sections of 5-μm-thickness were deparaffinized, rehydrated using the Automated Staining System AS-2 (SN: 180.001.1015.119; Pathisto), and antigen retrieval was performed in citrate buffer (pH 6.0, 82% 10-mM sodium citrate plus 18% of 10 mM citric acid) or in EDTA buffer (pH 8.0, 1 mM EDTA, 0.05% Tween 20) for 25 min at 110°C using Decloaking Chamber (SN: DG12-220-0134; Biocare Medical). At least 20 independent sections per genotype were histologically scored. For immunofluorescence assay, the sections were washed with PBS and incubated in blocking solution (5% horse serum in PBS) for 1 h. Subsequently, the slides were incubated overnight at 4°C with the following primary antibodies diluted in PBS: 1:500 monoclonal anti–pan-cytokeratin−FITC (F0397; Sigma-Aldrich), 1:500 anti-vimentin antibody (ab92547; Abcam), and 1:100 Phospho-Stat3 (Tyr705) antibody D3A7 (9145S; Cell Signaling Technology). After washing in PBS, Alexa Fluor 555 goat antirabbit IgG (A21428; Invitrogen) diluted 1:250 in PBS was applied for 1 h at room temperature. The sections were washed in PBS and mounted with DAKO Fluorescent Mounting Medium (S3023; DAKO) containing 1:1,000 Hoechst 33342 (H1399; Molecular probes by Life Technologies). Images were captured with Leica DMI6000 B and analyzed using the LAS X software or with Zeiss Axio Scan.Z1 and processed with ZEN software. Immunohistochemistry was performed following the instruction of Mouse on Mouse (M.O.M.) Basic Kit (BMK-2202; Vector Laboratories) combined with the protocol of ImmPRESS-AP Anti-Rabbit Ig Reagent–Alkaline Phosphatase (MP-5401-15; Vector Laboratories). Briefly, deparaffinization and antigen retrieval were performed as previously described. M.O.M. kit was used 1:1,000 monoclonal anti–pan-cytokeratin−FITC (F0397; Sigma-Aldrich), Anti-Ki67 antibody (15580; Abcam), and 1:1,000 Anti-CD31 antibody (ab182981; Abcam). Detection of the signal was performed with the Vector Blue Substrate Kit (SK5300; Vector Laboratories) and DAB Substrate Kit (ab64238; Abcam) or AEC Substrate system (ab64252; Abcam). The slides were mounted with DAKO Fluorescent Mounting Medium (S3023; DAKO) and sealed with nail polish. Images were captured with Zeiss Axio Scan.Z1 and processed with ZEN software. For H&E, the slides were first deparaffinized for 20 min in Histo-Clear (D1620333; Chemie Brunschwig), and then rehydrated for 1 min each in 100%, 96%, 80%, and 70% ethanol and ddH_2_O. After 10 min in hematoxylin, the slides were washed for 30 min in tap water, 30 min in Scott Water (8 mM MgSO_4_ × 7H_2_O [M2643-500G; Sigma-Aldrich] and 24 mM NaHCO_3_ [S5761; Sigma-Aldrich]), and 1 min in eosin (0.2%, 41-6660-00; MEDITE) containing three drops of CH_3_COOH. Subsequently, the slides were dehydrated and finally placed for 20 min in xylol (103746; EBIS). Then, mounting was performed using Eukitt Quick hardening mounting medium (03989; Fluka). Images were captured with Zeiss Axio Scan.Z1 and processed with ZEN software.

### Quantifications

Quantification of Vimentin^+^ and pan-cytokeratin^+^ cells was performed using a machine learning bio-image analysis software: QuPath (Bankhead et al, 2017). The software was trained to identify tumor cells, stromal cells, EMT cells, and false positives. Quantification of CD31 signal and ki67^+^ cells over pan-cytokeratin area was performed with CellProfiler (www.cellprofiler.org), a cell image analysis software. Proliferation rate was calculated by identification of tumor area and subsequent count of proliferating nuclei within the mask made out of the tumor. Relative enrichment of vessels within the tumors was performed by identification of tumor and vessel areas and subsequent quantification with an inverse mask out of tumor area for CD31^+^ signal. The correlation of the number of vessels with the CD31^+^ signal was statistically significant, out of three tumors quantified (Pearson r = 0.9990; two-tailed *P* = 0.0289). For the quantification of vimentin staining, we have used five different *Lgr5-CreER*^*T2*^*;LSL-ALK*^*F1174L*^*;LSL-Kras*^*G12D*^ mice from which we analyzed 21 independent tumors (one section per tumor) and five different *Lgr5-CreER*^*T2*^*;LSL-ALK*^*F1174L*^ mice from which we analyzed 12 independent tumors. For CD 31 quantification, we used 60 independent *Lgr5-CreER*^*T2*^*;LSL-ALK*^*F1174L*^*;LSL-Kras*^*G12D*^ tumors (one section per tumor) and 28 independent *Lgr5-CreER*^*T2*^*;LSL-ALK*^*F1174L*^ tumors (one section per tumor). For Ki67 staining, we used 70 independent *Lgr5-CreER*^*T2*^*;LSL-ALK*^*F1174L*^*;LSL-Kras*^*G12D*^ tumors (one section each) and 46 independent *Lgr5-CreER*^*T2*^*;LSL-ALK*^*F1174L*^ tumors (one section per tumor).

### Statistical analysis

Statistical analysis and graphical representation were performed with GraphPad Prism 5 software. The n reported in the figures or figure legends always refer to the number of mice used for each analysis. Column analysis was always performed with *t* test or Mann–Whitney test accordingly if the group of data considered in examination had a normal distribution or not (D’Agostino & Pearson omnibus normality test). Log-rank (Mantel–Cox) test was performed for statistical analysis of survivals.

### DNA extraction from paraffin tissue and sequencing of Alk exon 23

Paraffin-embedded cSCC from DMBA/TPA–treated mice were kindly gifted by the Laboratory of Prof. Sabine Werner. A few sections of tumors were collected in an Eppendorf tube together with 500 μl lysis buffer, which was incubated at 95°C for 10 min, and then centrifuged at 4°C, 20.817*g* for 5 min and the paraffin was removed. DNA extraction was performed as described above. Exome 23 of Alk was then amplified using KAPA Taq ReadyMix with dye (KK1024; Kapa Biosystems) with specific primers (msAlk-ex23_fw: CTATGCATCGCCCCAGGAAG, msAlk-ex23_Rv: GGCTGACTCCCAGGAGCCCA; MT = 60°C), and amplicons were sent for Sanger sequencing to Microsynth. Sequencing results were analyzed with Sequencer 5.1 (Genecode) and compared with reference sequence downloaded from Ensembl (www.ensembl.org).

### FACS sorting and RNA sequencing

FACS isolation of ear tumor cells and normal ear keratinocytes was performed as previously described (Nassar et al, 2015). mRNA from sorted cells was extracted using the RNeasy Plus Micro Kit (74034; QIAGEN) according to the manufacturers’ instructions. Biological replicates from nine independent mice, three Lgr5-CreER^T2^;LSL-ALK^F1174L^, three Lgr5-CreER^T2^;LSL-ALK^F1174L^;Kras^G12D^, and three Cre^−^ littermates, were sent to the Functional Genomic Center Zurich for RNA sequencing. RNA-seq-poly-A strategy was used to build the libraries, and Illumina Novaseq 6000 (Illumina) was used for sequencing. For following analysis, genes were selected based on *P*-value less than 0.01, and Log_2_ expression fold change is either above 0.5 or less than −0.5. GO and KEGG signaling pathways analysis was performed by using Database for Annotation, Visualization, and Integrated Discovery (DAVID) bioinformatics web tool. Gene Set Enrichment Analysis was performed against the MSigDB c5.bp.v6.2 gene set and clustering of enriched biological processes was performed using Cytoscape’s EnrichmentMap tool (version 3.2.0). Graphical visualization of RNA sequencing data was performed using R (version 3.5.0) and displayed in terms of Z-score values. RNA-seq data were deposited and made publicly available on the Gene Expression Omnibus (GSE147642).

### Cell culture

HEK293T cell line (ATCC CRL-3216) was cultured in DMEM Low Glucose w/L-Glutamine w/Sodium Pyruvate medium (L0060-500; Dominique Deutscher) enriched with 1.75*g* D-(+)-glucose (G8270; Sigma-Aldrich), 10% FCS (S181B-500; Dominique Deutscher), 1% L-glutamine (25030024; Gibco), and 1% sodium pyruvate (11360-039; Gibco) and maintained in a humidified incubator at 37°C, 5% CO_2_. Transient transfection was performed using Lipofectamine 2000 (11668-019; Thermo Fisher Scientific) according to the manufacturers’ protocol. The following plasmids were used: *pCMV6-ALKF*^*1174L-*^*Myc-DDK-tagged* (RC400189; OriGene) and pCMV6-Entry (PS100001; OriGene). 48 h post-transfection, protein extraction was performed.

### Western blot

Proteins were extracted using radio-immunoprecipitation assay (RIPA) lysis buffer (89900; Thermo Fisher Scientific) containing cOmplete ULTRA protease inhibitor (05892970001; Roche) and PhosSTOP (4906837001; Roche) and Phosphatase Inhibitor Cocktail 2 and 3 (P5726, P0044; Sigma-Aldrich). Cell lysates were centrifuged for 30 min at 20.817*g* at 4°C and supernatant was collected. Protein quantification was performed by Pierce BCA assay according to the manufacturers’ instructions (23227; Thermo Fisher Scientific). 30 μg of protein lysates mixed with Laemmli sample buffer (161-0747; Bio-Rad) was loaded into Precast Tris–HCl gels, 4–20% (456-8093; Bio-Rad). Trans-Blot Turbo Transfer System (690BR013492; Bio-Rad) was used to blot the gel onto polyvinylidene fluoride (PVDF) membranes (170-4156; Bio-Rad) and subsequently blocked for 1 h in 5% milk in TBS-T (TBS with 0.1% Tween [P4780-500ML; Sigma-Aldrich]). Overnight application of the following antibodies diluted in TBS-T was used: 1:500 Phospho-ALK (Tyr1604) Antibody (3341; Cell Signaling Technology), 1:1,000 Phospho-STAT3 (T705) Antibody (9131; Cell Signaling Technology), 1:1,000 Phospho-STAT3 (S727) Antibody (9134; Cell Signaling Technology), 1:2,000 in GAPDH Loading Control Monoclonal Antibody (MA5-15738; Thermo Fisher Scientific), and 1:5,000 Monoclonal anti-α-Tubulin (T-5168; Sigma-Aldrich). The membranes were washed in TBS-T and then incubated for 1 h at room temperature with the following secondary antibodies (1:2,000 in 5% milk in TBS-T): HRP anti-rabbit IgG (410406; BioLegend) and goat anti-mouse IgG HRP (405306; BioLegend). The signal was detected by application of Clarity Western ECL Substrate (170-5060; Bio-Rad) using Fusion FX VILBER LOURMAT (12-200168).

At least four independent tumors per genotype were subjected to Western blot analysis.

## Supplementary Material

Reviewer comments
