# Supplementary material for: Oncogenic ALKF1174L drives tumorigenesis in cutaneous squamous cell carcinoma
Source: Life Sci Alliance. 2020 Apr 20;3(6):e201900601. doi: 10.26508/lsa.201900601 (PMC7184028; doi:10.26508/lsa.201900601)
Supplement: Reviewer comments [file LSA-2019-00601_review_history.pdf]

Oncogenic ALKF1174L drives tumorigenesis in cutaneous squamous cell carcinoma

Marco Gualandi, Maria Iorio, Olivia Engeler, André Serra-Roma, Giuseppe Gasparre, Johannes Schulte, Daniel Hohl and Olga Shakhova
DOI: 10.26508/lsa.201900601

Corresponding author(s): Dr. Olga Shakhova (University of Zurich)

Review timeline:

Submission Date:	2019-11-07
Editorial Decision:	2019-12-20
Revision Received:	2020-02-27
Editorial Decision:	2020-03-23
Revision Received:	2020-03-30
Editorial Decision:	2020-03-31
Revision Received:	2020-04-07
Accepted:	2020-04-07

Scientific Editor: Andrea Leibfried

Transaction Report:

(Note: With the exception of the correction of typographical or spelling errors that could be a source of ambiguity, letters and reports are not edited. The original formatting of letters and referee reports may not be reflected in this compilation.)

No full Peer Review Process File is available with this article, as the authors have chosen not to make the review process public in this case.

1st Editorial Decision 20 December 2019

Re: Life Science Alliance manuscript #LSA-2019-00601-T

Dr. Olga Shakhova
University of Zurich
Oncology
Wagistrasse 14
Schlieren 8952
Switzerland

Dear Dr. Shakhova,

Thank you for submitting your manuscript entitled "Oncogenic ALKF1174L drives tumorigenesis in cutaneous squamous cell carcinoma" to Life Science Alliance. The manuscript was assessed by expert reviewers, whose comments are appended to this letter. We were expecting a third review on your work. Should this review still get submitted within a week, I will forward it to you.

As you will see, the reviewers appreciate your analyses, but think that some issues need to get addressed. We would thus like to invite you to submit a revised version, addressing the individual points raised by the reviewers. I will not revert this decision even if the third review is more negative on your work, but will outline to you how to address the additional comments that may still arrive.
Regarding the two reports at hand, the reviewers point to missing controls that should get added. Some other issues noted can get addressed by text changes. Importantly, however, both reviewers think that the link to STAT3 is rather weak. Please either provide stronger evidence following the constructive input the two reviewers provided or remove this part from your manuscript.

To upload the revised version of your manuscript, please log in to your account: https://lsa.msubmit.net/cgi-bin/main.plex
You will be guided to complete the submission of your revised manuscript and to fill in all necessary information. Please get in touch in case you do not know or remember your login name.

We would be happy to discuss the individual revision points further with you should this be helpful.

While you are revising your manuscript, please also attend to the below editorial points to help expedite the publication of your manuscript. Please direct any editorial questions to the journal office.

The typical timeframe for revisions is three months. Please note that papers are generally considered through only one revision cycle, so strong support from the referees on the revised version is needed for acceptance.

When submitting the revision, please include a letter addressing the reviewers' comments point by point.

We hope that the comments below will prove constructive as your work progresses.

Thank you for this interesting contribution to Life Science Alliance. We are looking forward to receiving your revised manuscript.

Sincerely,

Andrea Leibfried, PhD
Executive Editor
Life Science Alliance
Meyerhofstr. 1
69117 Heidelberg, Germany
t +49 6221 8891 502
e a.leibfried@life-science-alliance.org
www.life-science-alliance.org

A. THESE ITEMS ARE REQUIRED FOR REVISIONS

-- A letter addressing the reviewers' comments point by point.

-- An editable version of the final text (.DOC or .DOCX) is needed for copyediting (no PDFs).

-- High-resolution figure, supplementary figure and video files uploaded as individual files: See our detailed guidelines for preparing your production-ready images, http://www.life-science-alliance.org/authors

-- Summary blurb (enter in submission system): A short text summarizing in a single sentence the study (max. 200 characters including spaces). This text is used in conjunction with the titles of papers, hence should be informative and complementary to the title and running title. It should describe the context and significance of the findings for a general readership; it should be written in the present tense and refer to the work in the third person. Author names should not be mentioned.

B. MANUSCRIPT ORGANIZATION AND FORMATTING:

Full guidelines are available on our Instructions for Authors page, http://www.life-science-alliance.org/authors

We encourage our authors to provide original source data, particularly uncropped/-processed electrophoretic blots and spreadsheets for the main figures of the manuscript. If you would like to add source data, we would welcome one PDF/Excel-file per figure for this information. These files will be linked online as supplementary "Source Data" files.

IMPORTANT: It is Life Science Alliance policy that if requested, original data images must be made available. Failure to provide original images upon request will result in unavoidable delays in publication. Please ensure that you have access to all original microscopy and blot data images before submitting your revision.

2nd Editorial Decision 23 March 2020

RE: Life Science Alliance Manuscript #LSA-2019-00601-TR

Dr. Olga Shakhova
University of Zurich
Oncology
Wagistrasse 14
Schlieren 8952
Switzerland

Dear Dr. Shakhova,

Thank you for submitting your revised manuscript entitled "Oncogenic ALKF1174L drives tumorigenesis in cutaneous squamous cell carcinoma". As you will see, while the reviewers appreciate the introduced changes, some concerns remain. We would thus like to invite you to submit a final version of your manuscript, addressing the remaining concerns as well as some formatting requirements. While doing so will not require additional experimental work, we are aware that even more minor revisions may take some time given the current pandemic/COVID-19/SARS-CoV-2 situation, and we are happy to give you the time you need to address the remaining issues:

- Please address the remaining reviewer concerns - as mentioned before it is OK to remove the Stat3 data in order to do so
Also,
- Please make sure that the author order in our submission system matches the one depicted in the manuscript file
- Please link your ORCID iD to your profile in our submission system, you should have received an email with instructions on how to do so
- Please upload all figures, including supplementary figures, as individual files
- Please upload the manuscript text in docx format
- Please add callouts in the manuscript text to figures: Fig. 2c; Fig. 3a,j; Fig. 4 b,c; Suppl. Fig 1f; Suppl. Fig 4d; Suppl. Fig S6d
- Please add the panel f to figure S1 (currently missing, but described in figure legend)
- Please list 10 authors et al in your reference list
- Please deposit the RNA-seq data in a repository and provide the accession code in the M&M section of your article
- Please indicate the parts of the image used for the higher magnification in Figure 3k, 3o, 4g
- Please add a scale bar to figure S1e

If you are planning a press release on your work, please inform us immediately to allow informing our production team and scheduling a release date.

To upload the final version of your manuscript, please log in to your account: https://lsa.msubmit.net/cgi-bin/main.plex
You will be guided to complete the submission of your revised manuscript and to fill in all necessary information. Please get in touch in case you do not know or remember your login name.

To avoid unnecessary delays in the acceptance and publication of your paper, please read the following information carefully.

A. FINAL FILES:

These items are required for acceptance.

-- An editable version of the final text (.DOC or .DOCX) is needed for copyediting (no PDFs).

-- High-resolution figure, supplementary figure and video files uploaded as individual files: See our detailed guidelines for preparing your production-ready images, http://www.life-science-alliance.org/authors

-- Summary blurb (enter in submission system): A short text summarizing in a single sentence the study (max. 200 characters including spaces). This text is used in conjunction with the titles of papers, hence should be informative and complementary to the title. It should describe the context and significance of the findings for a general readership; it should be written in the present tense and refer to the work in the third person. Author names should not be mentioned.

B. MANUSCRIPT ORGANIZATION AND FORMATTING:

Full guidelines are available on our Instructions for Authors page, http://www.life-science-alliance.org/authors

We encourage our authors to provide original source data, particularly uncropped/-processed electrophoretic blots and spreadsheets for the main figures of the manuscript. If you would like to add source data, we would welcome one PDF/Excel-file per figure for this information. These files will be linked online as supplementary "Source Data" files.

Submission of a paper that does not conform to Life Science Alliance guidelines will delay the acceptance of your manuscript.

It is Life Science Alliance policy that if requested, original data images must be made available to the editors. Failure to provide original images upon request will result in unavoidable delays in publication. Please ensure that you have access to all original data images prior to final submission.

The license to publish form must be signed before your manuscript can be sent to production. A link to the electronic license to publish form will be sent to the corresponding author only. Please take a moment to check your funder requirements.

Reviews, decision letters, and point-by-point responses associated with peer-review at Life Science Alliance will be published online, alongside the manuscript. If you do want to opt out of having the reviewer reports and your point-by-point responses displayed, please let us know immediately.

Thank you for your attention to these final processing requirements.

Thank you for this interesting contribution, we look forward to publishing your paper in Life Science Alliance.

Sincerely,

© Life Science Alliance

Andrea Leibfried, PhD
Executive Editor
Life Science Alliance
Meyerhofstr. 1
69117 Heidelberg, Germany
t +49 6221 8891 502
e a.leibfried@life-science-alliance.org
www.life-science-alliance.org

3rd Editorial Decision 31 March 2020

RE: Life Science Alliance Manuscript #LSA-2019-00601-TRR

Dr. Olga Shakhova
University of Zurich
Oncology
Wagistrasse 14
Schlieren 8952
Switzerland

Dear Dr. Shakhova,

Thank you for submitting your revised manuscript entitled "Oncogenic ALKF1174L drives tumorigenesis in cutaneous squamous cell carcinoma". We would be happy to publish your paper in Life Science Alliance pending final revisions necessary:

- The section relating to figure 5 needs re-writing in my view.

1. Currently, you state:
(...) Despite the fact that ALKF1174L Stat3fl/fl Lgr5-CreERT2 showed prolonged tumor-free survival and progression-free survival as compared to ALKF1174L Lgr5-CreERT2 mice, they still exhibited tumor formation (Fig. 5d,e). (...)

=> I think it would make more sense to write:
ALKF1174L Stat3fl/fl Lgr5-CreERT2 showed prolonged tumor-free survival and progression-free survival as compared to ALKF1174L Lgr5-CreERT2 mice. However, tumor formation still occurred (Fig. 5d,e).

2. Currently, you state:
(...) We next evaluated the percentage of the recombination and counted the number of HF either positive or negative for p-Stat3 protein (all WT HF were devoid of p-Stat3). While the vast majority of HF in ALKF1174L Lgr5-CreERT2 mice showed readily detectable p-Stat3 protein, less than 50 % of HF were positive for p-Stat3 in ALKF1174L Stat3fl/fl Lgr5-CreERT2 mice (Fig. 5h and Supp. Fig. 6d). (...)

=> I think you need to add a sentence here to link this result to the observed tumor incidence, speculating that delayed tumor formation may be due to cells deficient of STAT3 and expressing ALKF1174L likely stop proliferating or have reduced cell viability. Those HF expressing p-STAT3 due to the low recombination frequency likely give rise to the tumors observed.

3. Currently, you state:
(...)Given the fact that SCC development occurred only in presence of Stat3, we conclude that Stat3 is indispensable for ALKF1174L -mediated tumor formation. (...)

=> I don't think you can state this based on the data provided, as the cKO mice develop tumors and it is unclear whether this is indeed due to the observed recombination frequency (no Stat3 deficiency established). You would probably need to cross the ALKF1174L mice with Stat3 KO mice to be able to potentially draw such a firm conclusion.

© Life Science Alliance

Please rewrite.

- the abstract needs to also further tone down the conclusion on Stat3, I'd suggest to write:
Finally, we demonstrate that Stat3 is a key downstream effector of ALKF1174L and likely plays a role in ALKF1174L-driven cSCC tumorigenesis.

If you are planning a press release on your work, please inform us immediately to allow informing our production team and scheduling a release date.

To upload the final version of your manuscript, please log in to your account: https://lsa.msubmit.net/cgi-bin/main.plex
You will be guided to complete the submission of your revised manuscript and to fill in all necessary information. Please get in touch in case you do not know or remember your login name.

To avoid unnecessary delays in the acceptance and publication of your paper, please read the following information carefully.

A. FINAL FILES:

These items are required for acceptance.

-- An editable version of the final text (.DOC or .DOCX) is needed for copyediting (no PDFs).

-- High-resolution figure, supplementary figure and video files uploaded as individual files: See our detailed guidelines for preparing your production-ready images, http://www.life-science-alliance.org/authors

-- Summary blurb (enter in submission system): A short text summarizing in a single sentence the study (max. 200 characters including spaces). This text is used in conjunction with the titles of papers, hence should be informative and complementary to the title. It should describe the context and significance of the findings for a general readership; it should be written in the present tense and refer to the work in the third person. Author names should not be mentioned.

B. MANUSCRIPT ORGANIZATION AND FORMATTING:

Full guidelines are available on our Instructions for Authors page, http://www.life-science-alliance.org/authors

We encourage our authors to provide original source data, particularly uncropped/-processed electrophoretic blots and spreadsheets for the main figures of the manuscript. If you would like to add source data, we would welcome one PDF/Excel-file per figure for this information. These files will be linked online as supplementary "Source Data" files.

Submission of a paper that does not conform to Life Science Alliance guidelines will delay the acceptance of your manuscript.

**It is Life Science Alliance policy that if requested, original data images must be

made available to the editors. Failure to provide original images upon request will result in unavoidable delays in publication. Please ensure that you have access to all original data images prior to final submission.**

The license to publish form must be signed before your manuscript can be sent to production. A link to the electronic license to publish form will be sent to the corresponding author only. Please take a moment to check your funder requirements.

Reviews, decision letters, and point-by-point responses associated with peer-review at Life Science Alliance will be published online, alongside the manuscript. If you do want to opt out of having the reviewer reports and your point-by-point responses displayed, please let us know immediately.

Thank you for your attention to these final processing requirements. Please revise and format the manuscript and upload materials within 7 days.

Thank you for this interesting contribution, we look forward to publishing your paper in Life Science Alliance.

Sincerely,

Andrea Leibfried, PhD
Executive Editor
Life Science Alliance
Meyerhofstr. 1
69117 Heidelberg, Germany
t +49 6221 8891 502
e a.leibfried@life-science-alliance.org
www.life-science-alliance.org

4th Editorial Decision 7 April 2020

RE: Life Science Alliance Manuscript #LSA-2019-00601-TRRR

Dr. Olga Shakhova
University of Zurich
Oncology
Wagistrasse 14
Schlieren 8952
Switzerland

Dear Dr. Shakhova,

Thank you for submitting your Research Article entitled "Oncogenic ALKF1174L drives tumorigenesis in cutaneous squamous cell carcinoma". I appreciate the introduced changes and it is a pleasure to let you know that your manuscript is now accepted for publication in Life Science Alliance. Congratulations on this interesting work.

The final published version of your manuscript will be deposited by us to PubMed Central upon online publication.

Your manuscript will now progress through copyediting and proofing. It is journal policy that authors provide original data upon request.

Reviews, decision letters, and point-by-point responses associated with peer-review at Life Science Alliance will be published online, alongside the manuscript. If you do want to opt out of having the reviewer reports and your point-by-point responses displayed, please let us know immediately.

IMPORTANT: If you will be unreachable at any time, please provide us with the email address of an alternate author. Failure to respond to routine queries may lead to unavoidable delays in publication.

Scheduling details will be available from our production department. You will receive proofs shortly before the publication date. Only essential corrections can be made at the proof stage so if there are any minor final changes you wish to make to the manuscript, please let the journal office know now.

DISTRIBUTION OF MATERIALS:
Authors are required to distribute freely any materials used in experiments published in Life Science Alliance. Authors are encouraged to deposit materials used in their studies to the appropriate repositories for distribution to researchers.

You can contact the journal office with any questions, contact@life-science-alliance.org

Again, congratulations on a very nice paper. I hope you found the review process to be constructive and are pleased with how the manuscript was handled editorially. We look forward to future exciting submissions from your lab.

Sincerely,

Andrea Leibfried, PhD
Executive Editor
Life Science Alliance
Meyerhofstr. 1
69117 Heidelberg, Germany
t +49 6221 8891 502
e a.leibfried@life-science-alliance.org
www.life-science-alliance.org
